# Single-cell transcriptional and functional analysis of dopaminergic neurons in organoid-like cultures derived from human fetal midbrain

**DOI:** 10.1242/dev.200504

**Published:** 2022-12-08

**Authors:** Marcella Birtele, Petter Storm, Yogita Sharma, Janko Kajtez, Jenny Nelander Wahlestedt, Edoardo Sozzi, Fredrik Nilsson, Simon Stott, Xiaoling L. He, Bengt Mattsson, Daniella Rylander Ottosson, Roger A. Barker, Alessandro Fiorenzano, Malin Parmar

**Affiliations:** ^1^ Developmental and Regenerative Neurobiology, Wallenberg Neuroscience Center, and Lund Stem Cell Centre, Department of Experimental Medical Science, Lund University, Lund 223 62, Sweden; ^2^ Department of Clinical Neuroscience and Wellcome-MRC Cambridge Stem Cell Institute, University of Cambridge, Cambridge CB2 0PY, UK; ^3^ Regenerative Neurophysiology, Wallenberg Neuroscience Center, Lund Stem Cell Center, Department of Experimental Medical Science, Lund University, Lund 223 62, Sweden

**Keywords:** 3D cultures, Dopamine Neurons, Human fetal dopamine neuron, Neurobiology, Parkinson's disease

## Abstract

Significant efforts are ongoing to develop refined differentiation protocols to generate midbrain dopamine (DA) neurons from pluripotent stem cells for application in disease modeling, diagnostics, drug screening and cell-based therapies for Parkinson's disease. An increased understanding of the timing and molecular mechanisms that promote the generation of distinct subtypes of human midbrain DA during development will be essential for guiding future efforts to generate molecularly defined and subtype-specific DA neurons from pluripotent stem cells. Here, we use droplet-based single-cell RNA sequencing to transcriptionally profile the developing human ventral midbrain (VM) when the DA neurons are generated (6-11 weeks post-conception) and their subsequent differentiation into functional mature DA neurons in primary fetal 3D organoid-like cultures. This approach reveals that 3D cultures are superior to monolayer conditions for their ability to generate and maintain mature DA neurons; hence, they have the potential to be used for studying human VM development. These results provide a unique transcriptional profile of the developing human fetal VM and functionally mature human DA neurons that can be used to guide stem cell-based therapies and disease modeling approaches in Parkinson's disease.

## INTRODUCTION

Dopamine (DA) neurons in the ventral midbrain (VM) are diverse and consist of several anatomically defined subtypes with distinct projection targets and functions (Björklund and Dunnett, 2007). For example, the A9 DA neurons in the substantia nigra pars compacta (SNc) are selectively lost in Parkinson's disease (PD). In contrast, nearby A10 DA neurons of the ventral tegmental area are more closely associated with aspects of emotion and reward processing, and thus are implicated in neuropsychiatric disease and addiction (Anderegg, Poulin, and Awatramani, 2015; Roeper, 2013; Grace, 2016). Both A9 and A10 DA neurons originate from the ventral mesencephalic floor plate (Ono et al., 2007), but it is not yet fully known how the different dopaminergic neuron populations arise and develop. Moreover, recent single-cell RNA-sequencing (scRNA-seq) studies of adult mouse midbrain have revealed a greater than expected molecular diversity in mature DA neurons, suggesting heterogeneity even within the anatomically defined DA clusters (Tiklová et al., 2019; La Manno et al., 2016; Poulin et al., 2014).

Whereas midbrain DA neurons from pluripotent stem cells (PSCs) have already successfully been used for applications such as disease modeling, drug screening and cell-based therapies for PD (Ambasudhan et al., 2011; Reinhardt et al., 2013; Kouroupi et al., 2017; Ke et al., 2020; Cooper et al., 2012; Doi et al., 2020; Piao et al., 2021), it is not yet possible to control the generation of distinct subtypes of midbrain DA neurons. A better understanding of human DA neuron specification and maturation during development is vital in these efforts. scRNA-seq represents a major technological advance in determining cell state and inferring developmental trajectories (Rosenberg et al., 2018; Cao et al., 2017), and it has already been applied to increase our understanding of DA neuron development (La Manno et al., 2016; Saunders et al., 2018; Kirkeby et al., 2017). However, owing to limited access to human fetal brain tissue, such investigations have almost exclusively been performed in mice (Poulin et al., 2014; Hook et al., 2018; Kee et al., 2017). One study used single-cell transcriptomics to compare DA lineage in mouse and human development, which revealed several points of similarity and crucial differences between species (La Manno et al., 2016), highlighting the importance of studying midbrain development using human tissue. Moreover, a primate-specific substantia nigra pars compacta neuronal population has recently been identified (Kamath et al., 2022).

In this study, we used droplet-based scRNA-seq to perform high-throughput transcriptional profiling of >20,000 cells in the human fetal midbrain at different developmental stages. Our analysis identified the cellular composition of the developing VM at the developmental stage when DA neurons are generated, and revealed the early emergence of distinct DA populations. To study more mature human DA neurons, we established a fetal VM tissue-based organoid-like 3D culture model that supported differentiation into functionally mature DA neurons. Using transcriptional profiling of >12,000 neurons, we identified one progenitor population and three molecularly postmitotic DA subclusters.

## RESULTS

### Human ventral midbrain development

**Fig. 1.**
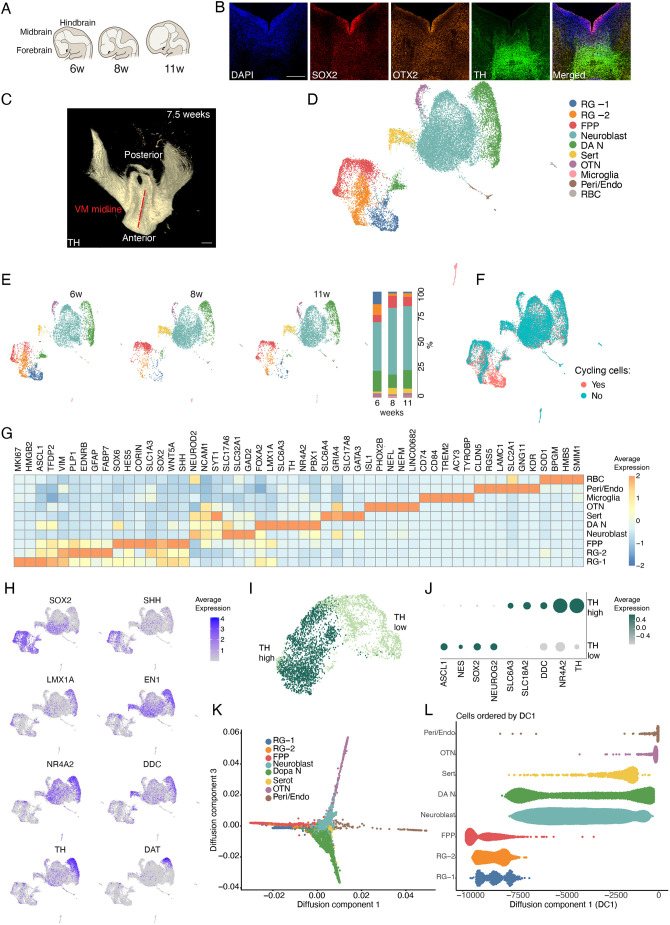
**sc-RNAseq of developing human fetal VM.** (A) Schematic representation of the sagittal view of forebrain, midbrain and hindbrain during human brain development (from 6 to 11 weeks post-conception). (B) Anterior view of a VM section at post-conception (PC) week 7.5. Immunohistochemistry of SOX2, OTX2 and TH. Scale bar: 250 µm. (C) iDISCO circuitry reconstruction obtained by mapping TH in a dorsal view of human fetal VM at PC week 7.5. Scale bar: 500 µm. (D) UMAP plot showing clustering of 23,483 analyzed cells from three separate fetuses (6, 8 and 11 weeks PC). Grouping of all the samples was initially performed to obtain a more accurate assignment of the different cell types. Cell type assignments based on La Manno et al. (2016) are indicated. (E) UMAP plots of cell clusters for each developmental stage of fetal VM analyzed and the fraction (%) of cells per cluster. (F) UMAP plots showing composite cell cycle scores of analyzed cells (S score and G2M score). (G) Heat map showing expression levels of indicated genes per cluster. Indicated genes are established markers for RG-1, RG-2, neuroblast, dopamine neurons (DA N), serotonergic neurons (Sert), oculomotor/trochlear nucleus neurons (OTN), microglia, pericytes/endothelial cells (Peri/Endo) and red blood cells (RBC). (H) Feature plots visualizing early and late DA markers across clusters. Gray and purple indicate expression levels. (I) UMAP plot showing DA subclusters determined by high and low *TH* expression, and (J) dot plot showing differentially expressed selected genes across high-TH and low-TH groups. (K) Diffusion map of diffusion components (DCs) 1 and 3 reconstructing post-mitotic cell maturation. Cells are colored according to cell type. (L) Pseudotemporal ordering of emerging cell types according to DC1.

We accessed human embryos at the stage when the DA neurons in the ventral midbrain arise, ranging from weeks 6 to 11 post-conception (PC) (Fig. 1A, Table S1). The mesencephalon, which is positioned in the medial part of the embryonic neural tube, was sub-dissected from the embryo. By 7.5 weeks in human embryos, SOX2 marks the proliferative ventricular zone (VZ), OTX2 is expressed from the ventricular to mantle zone and tyrosine hydroxylase (TH) labels DA post-mitotic neurons (Fig. 1B). iDISCO tissue clearing and light sheet microscopy (LSM) were used to visualize the TH-positive neurons and their projections within the VM at the developmental stage that the tissue was dissected and showed that the DA neurons, which are marked by TH expression, were found in high quantities and with already well-established projections at this time point (7.5 weeks PC) (Fig. 1C and Movies 1-3).

### Single-cell transcriptomics reveals the cellular composition of the developing human fetal VM

To determine the cellular composition of the developing VM at the molecular level, we performed droplet-based scRNA-seq on the dissected VM tissue from three separate fetuses (6, 8 and 11 weeks PC). After removing poor-quality cells (see Materials and Methods), 23,483 cells were retained for analysis: 6634 from 6 weeks, 8113 from 8 weeks and 8736 from 11 weeks. Uniform manifold approximation and projection (UMAP) graph-based clustering partitioned the cells into 10 distinct clusters (Fig. 1D). Three small but distinct groups were identified: microglia (pink cluster expressing *CD74*, *CD86*, *TREM2*, *ACY3* and *TYROBP*; 0.26-0.85% of total cell population across all embryos), pericytes/endothelial cells (brown cluster expressing *COL1A2*, *CLDN5*, *RGS5*, *LAMC1*, *GNG11* and *KDR*, as well as mesoderm-canonical markers such as CREB3L genes, 0.16-0.74% of total cell population across all embryos) and red blood cells (grey cluster expressing *SOD1*, *BPGM*, *HMBS*, *SLC2A1* and *SMIM1*; 0.38-0.59% of total cell population across all embryos). These cell types are expected contaminants after fetal tissue dissection. Most of the cells fell into the remaining seven clusters, which we assigned to major cell types through analysis of canonical and automated cell type annotation. We found two major clusters sharing key molecular features of radial glial (RG) cells, which we termed RG-1 (blue) and RG-2 (orange). Both clusters were characterized by the expression of *SOX2*, *PLP1*, *EDNRB* and *SOX9,* and they were proportionally reduced during later developmental stages of human VM embryos (Fig. 1D,E). RG-1 mainly included cycling RG cells in the ventricular zone and were distinguished by a highly proliferative signature (*TOP2A*, *MKI67* and *TFDP2*), as visualized by a composite cell cycle score (S-score and G2M-score), as well as by expression of the pro-neural gene *ASCL1* and the chromatin-associated gene *HMGB2* (Fig. 1F and Fig. S1A-C). RG-2, on the other hand, showed higher expression of canonical RG markers, including *FABP7* (also known as *BLBP1*) and *SLC1A3* (also known as *GLAST*), linking RG-1 with floor-plate progenitors (FPPs; red) (Fig. 1D,G and Fig. S1C). This last cluster was enriched with the expression of the morphogens *SHH*, *WNT5A*, *FOXA2*, *LMX1A* and *OTX2*, which define midbrain FP cells (Fig. 1D,G and Fig. S1C).

A predominant cluster (teal) was detected, mainly comprising post-mitotic cells with heterogeneous cell identities, referred to as Neuroblast (Fig. 1D,G). This cluster was characterized by the loss of proliferative markers (Fig. 1F and Fig. S1A,B) and expression of neuronal differentiation factor 2 (*NEUROD2*), the cytoskeletal marker neural cell-adhesion molecule 1 (*NCAM1*) and the synaptic marker *SYT1* (Fig. 1G). This cluster revealed an emerging cell type diversity, including the expression of genes associated with early dopaminergic (*FOXA2*, *EN2*, *NR4A2*, *LMX1A* and *TH*) and serotoninergic (Sert) (*GRIA4* and *GATA3*) neurons (Fig. 1D,G and Fig. S1C). We could also detect three types of mature neurons: serotonergic neurons (yellow group expressing *SLC6A4*, *GRIA4*, *SLC17A8* and *GATA3*; 2-6% of the total cell population across all embryos), oculomotor/trochlear nucleus (OTN) neurons (purple cluster expressing *ISL1*, *PHOX2B*, *NEFL*, *NEFM* and *LINC00682*; 1.54-3.48% of the total cell population across all embryos) and a much larger population of DA neurons [green cluster expressing *TH*, *DDC* and *NR4A2* (also known as *NURR1*); 12.63-19.5% of the total cell population across all embryos] (Fig. 1D,E,G). *TH* and DA transporter (*SLC6A3*, also known as *DAT*) expression were not homogeneously expressed in the DA cluster (Fig. 1H), prompting us to further investigate gene expression at the single-cell level within this population.

By iterating the resolution parameter on DA clusters from different embryos, we distinguished the most mature DA neuron population and identified two subclusters with high and low *TH* expression (TH^high^ and TH^low^, respectively, Fig. 1I). TH^low^ appeared to express canonical intermediate progenitor markers such as *HES6*, *DLL1*, *GADD45G*, *INSM1*, *NHLH1* and *NEUROG2*, and it was enriched in early DA markers such as *SOX2*, *EN1* and *SOX6*. TH^high^ population was instead characterized by higher expression of ion channels as well as by the expression of *DAT* and the synaptic vesicular transporter *SLC18A2* (also known as *VMAT2*), indicating a later DA differentiation stage (Fig. 1I,J and Fig. S1E). To investigate the relationships between single-cell expression profiles, differentiation states and developmental trajectories, we also plotted the data using diffusion maps, a noise-tolerant non-linear dimensionality reduction method that can reveal a global topology for the data based on local similarities between individual cells (Haghverdi et al., 2016). The resulting graph produced an intuitive developmental picture in which immature cells (RG and FPPs from the youngest embryos) and postmitotic differentiated cells occupied opposite ends of the DC1 axis, while neuroblasts cells localized to intermediate positions (Fig. 1K and Fig. S1F). Pseudo-temporal placing of cells from the resulting diffusion component identified the early emergence of dopamine neurons followed by the emergence of glutamatergic/serotonergic neurons and final OTN and perivascular cells (Fig. 1L).

**Fig. 2.**
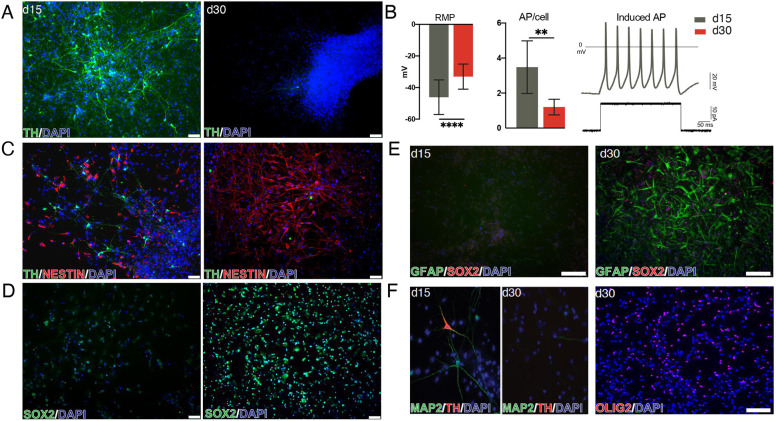
**2D culture condition affects DA neuron long-term maturation.** (A) Immunohistochemistry of TH in 2D culture at day (d) 15 and at d30. (B) Left: measurements from whole-cell patch-clamp recordings showing resting membrane potential (RMP) at d15 (*n*=25) and d30 (*n*=19). Recordings were performed on three different human embryos for each time point. Middle: measurements from whole-cell patch-clamp recordings showing mean number of action potentials (APs), per cell at d15 and at d30. Right: representative trace of multiple induced APs showing a neuronal profile of a patched cell at d15. **P<0.0025, ****P<0.0001. (C,D) Immunocytochemical images displaying increased levels of nestin (C) and SOX2 markers (D) at d30 compared with d15. (E) Immunocytochemical images displaying increases in GFAP at d30 compared with d15. (F) Left: immunocytochemical images displaying the neuronal marker MAP2 and TH, confirming the presence of both neurons and dopaminergic cells at d15. At d30, TH- and MAP2-positive cells were difficult to detect. Right: immunocytochemical images displaying OLIG2 expression at d30. Scale bars: 75 µm.

These data provide a time-resolved single-cell map of the developing human fetal VM and shows the emergence of neuronal DA populations at this stage of development. Further investigations into fetal VM at later stages of development would be highly desirable, but access to second and third trimester fetal tissue is very restrictive and cannot be easily used for studies of this type. Thus, to undertake transcriptional studies of functionally mature human DA neurons, we established primary cultures from human VM (hVM) collected from three fetuses (8, 9 and 10 weeks PC) (Fig. S2A,B, Table S1). After 2 weeks of differentiation, many TH^+^ cells with neuronal morphology were detected in these cultures (Fig. 2A). Whole-cell patch-clamp recordings on day 15 showed that these cells (*n*=25) had a resting membrane potential (RMP) in line with a neuronal identity (Fig. 2B and Fig. S2C,D, black arrowhead indicating neuronal morphology), and we confirmed that the cells could fire induced action potentials (APs) (Fig. 2B). At this time point, we found that 23.5% of the cells recorded were also able to spontaneously fire APs (Fig. S2E), suggesting that they had started a process of functional maturation *in vitro*. We attempted to analyze the neurons after an additional 2 weeks in culture to assess more mature DA neurons. However, cells with neuronal morphology were sparsely distributed at this stage (Fig. S2C, white arrowhead indicates non-neuronal morphology) and immunocytochemical analysis showed that very few TH^+^ neurons remained in the cultures (Fig. 2A,C,F). When cells were recorded at day 30, no inward Na^+^ and outward K^+^ voltage-dependent currents, induced APs or spontaneous APs were observed (*n*=19 cells) (Fig. 2B and Fig. S2D,E). The depletion of TH^+^ neurons over time in 2D culture was accompanied by an expansion of immature neuronal cells (NESTIN and SOX2) (Fig. 2C,D), and by the emergence of non-neuronal populations containing glial (GFAP) and oligodendrocyte (OLIG2) progenitors (Fig. 2E,F).

### 3D culture recapitulates functionally mature human DA neurons

**Fig. 3.**
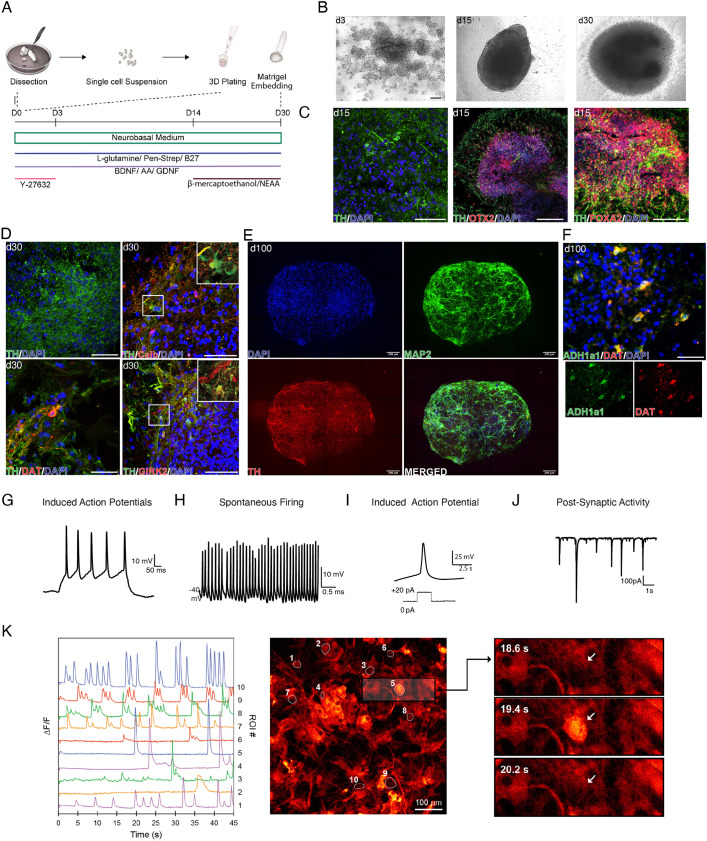
**3D culture environment allows for the differentiation and maturation of human fetal DA.** (A) Schematic overview of protocol and experimental design. (B) Representative bright-field images of 3D human fetal VM culture differentiation at different time points. Scale bar: 100 µm. (C) Cryosection of 3D human fetal VM culture at day 15 showing TH, OTX2, FOXA2 staining. Scale bars: 100 µm. (D) Cryosection of 3D human fetal VM culture at day 30 showing TH, DAT, CALB and GIRK2 staining. Scale bars: 100 µm. (E) 3D reconstruction of an image stack from a 80 µm optical section of DAPI, TH and MAP2 immunohistochemistry at day 100. Scale bars: 200 µm. (F) Immunohistochemistry of 3D human fetal VM culture at day 100 showing DAPI, DAT and ALDH1A1-positive neurons. (G) Representative trace of induced action potentials (APs) at day 30 from 3D cultures indicative of mature neuronal profile. (H) Representative trace of spontaneous firing indicative of a possible DA neuronal profile. (I) Representative trace of induced APs elicited by a small step of current injection indicative of a DA neuronal profile. (J) Representative trace of postsynaptic activity indicative of active neuronal network connections in the 3D system. (K) Differential fluorescence intensity profile as a function of time for 3D hVM cultures at day 100 expressing MAP2–GCamP3 (left); fluorescence image with segmented regions of interest corresponding to individual cells (middle). Scale bar: 100 µm. Three timeframes displaying the change in intercellular fluorescence intensity for two cells indicated by arrows (right).

We hypothesized that fetal hVM cells cultured in 3D organoid-like structures could better maintain DA neuron sources from the fetal brain in long-term cultures. We therefore induced self-aggregation of 70,000 fetal VM cells using low attachment U-bottom plates (Fig. 3A). A cluster assembly was formed by day 3, followed by complete 3D structure formation at day 15 (Fig. 3B). Immunohistochemical analysis using antibodies for the mesodiencephalic FP/DA progenitor markers FOXA2 and OTX2 confirmed the midbrain identity of the cells (Fig. 3C). Immunohistochemical analysis revealed the presence of TH^+^ neurons at day 15, similar tour observations in 2D primary cultures (Fig. 3C). However, in stark contrast to monolayer culture, the TH neurons in 3D structures were maintained at day 30 (Fig. 3D,E, Fig. S3A). On day 30, we also found the expression of mature DA markers, such as DAT, dopa decarboxylase (AADC) (Fig. S3A), calcium-binding protein 1 (CALB1) and the G-protein-regulated inward-rectifier potassium channel (GIRK2) (Fig. 3D), indicating a mature subtype-specific identity of DA neurons in these cultures. Expression of TH, DAT and ALDH1A1 was maintained in 3D cultures analyzed for up to 100 days (Fig. 3E,F). Whole-cell patch-clamp recordings at day 30 revealed that most cells (17/24) could fire multiple mature APs (Fig. 3G and Fig. S3B). One-third of the cells also showed spontaneous firing (8/24; Fig. 3H) with continuous tonic like discharges reported in *in vivo* DA neurons (Chergui et al., 1993; Dreyer et al., 2010). Multiple or single APs generated upon current injection displayed a slow after-hyperpolarization potential (8/24; Fig. 3I), which is typical of DA neurons (Nedergaard, 2004), and an AP width similar to that reported in primate and rodent neurons (Nedergaard, 1999). Postsynaptic activity was also detected in 10% of the cells (Fig. 3J). By performing both confocal spinning disk imaging (Fig. 3E) and iDISCO (Fig. S3C and Movies 4), we obtained an anatomical 3D reconstruction of the complex DA neuronal circuitry and bundle connections within 3D fetal structures, showing that these DA neurons could be stably maintained for up to 3 months *in vitro*. To further assess whether this intricate DA network corresponded to a functional and active neuronal map, we performed calcium imaging of MAP2-GcaMP3-labeled neurons from 3D VM cultures (Fig. 3K and Movie 5) at 3 months. The presence of calcium waves with different kinetics in MAP2^+^ cells indicated active neuronal signaling (Fig. 3K), demonstrating that healthy and functionally mature neurons were maintained in these 3D fetal cultures over time.

### scRNA-seq identifies cell diversity and DA molecular identities in 3D human fetal VM organoid-like cultures

**Fig. 4.**
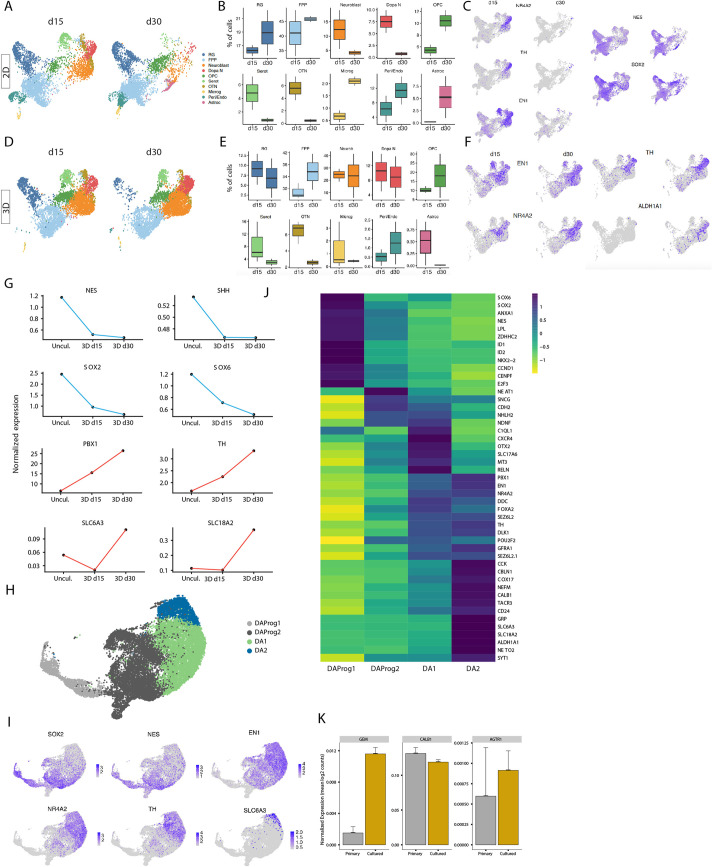
**scRNA-seq captures the molecular diversity of DA neurons within 3D fetal hVM culture.** (A) UMAP plot showing 2D human fetal VM cultures at day (d) 15 and at d30. (B) Box plots of percentage of cells belonging to each identified cluster at d15 and d30 of 2D human fetal VM cultures. Upper box limit represents maximum value, lower box limit minimum value, horizontal line mean value and whiskers s.e.m. (C) Feature plots visualizing early and late DA markers across clusters at d15 and d30 of 2D human fetal VM cultures. Gray and purple colors indicate expression levels. (D) UMAP plot showing 3D human fetal VM cultures at d15 and at d30. (E) Box plots of the percentage of cells belonging to each identified cluster at d15 and d30 of 3D human fetal VM cultures. (F) Feature plots visualizing early and late DA markers across clusters at d15 and d30 of 2D human fetal VM cultures. Gray and purple colors indicate expression levels. (G) Pseudotemporal expression pattern of early and late DA markers in hVM and 3D hVM cultures at d15 and d30. (H) UMAP plot showing DA sub-clusters from scRNA-seq dataset of 3D human fetal VM cultures (d15 and d30). Selection of DA sub-clusters was performed based on cell-type assignment referencing the dataset published by La Manno et al. (2016) combined with label transfer from the uncultured dataset (see Fig. 1). (I) Feature plots visualizing early and late DA markers in DA sub-clusters of 3D human fetal VM cultures (d15 and d30). Gray and purple colors indicate expression levels. (J) Heatmap showing differentially expressed genes and manually selected markers in four DA neuron subclusters (DA-Prog1, DA-Prog2, DA-1 and DA-2). (K) Expression of primate specific genes in primary and cultured dopamine neurons. Data are mean±s.e.m.

Histological and functional analysis showed that DA neurons differentiated over time and that mature DA neurons were present in the long-term 3D cultures of human fetal VM. To comprehensively characterize the cellular composition of these 3D cultures, we performed scRNA-seq at d15 and d30 (Fig. 4D-F) and compared them with scRNA-seq of 2D cultures (Fig. 4A-C).

scRNA-seq revealed a high similarity of the human DA neurons after 15 days in 2D or 3D cultures but the ability to maintain mature DA neurons in the organoids also allowed for transcriptional profiling at a later timepoint. This analysis revealed that although the relative proportion of Sert and OTN neuron populations decreased over time in this 3D model, the DA neuron cluster was preserved and became the predominant neuronal cell type (Fig. 4D-F). Feature plots of well-established early and late DA neurogenesis markers visualized a robust DA population (Fig. 4F). Although RG and FP populations did not vary significantly over time, microglia were almost absent after 15 days in 3D culture (Fig. 4D-F). Moreover, whereas oligodendrocyte progenitors emerged as a distinct cluster, astrocytes, which were previously captured as an expanding population in 2D culture, were almost absent at both timepoints (Fig. 4D-F).

We next pseudo-temporally reconstructed the expression of the early and late DA markers detected in the fetal VM tissue (Fig. 1I) and the 3D cultures. The integrated scRNA-seq data showed that expression levels of late and mature DA markers such as *PBX1*, *TH*, *SLC6A3* and *VMAT2* increased over time with a corresponding decrease in *NES*, *SHH*, *SOX2* and *SOX6* (Fig. 4G). Overall, these findings validate our 3D culture system as a functional model that is able to mimic later stages of fetal DA neuron development *in vitro*.

The large number of immature and mature DA neurons present (*n*=12,600, identified using label transfer from uncultured neurons and publicly available data, details in the Materials and Methods) in fetal 3D cultures enabled us to perform a high-resolution analysis of DA neuron diversity at the single-cell level. We employed graph-based clustering to subcluster the DA population, which was found to be segregated into four clusters (Fig. 4H), with cohesion and separation supported by silhouette analysis (Fig. S4B). Visualization of canonical markers revealed two immature populations sharing molecular features of DA progenitors (*NES*, *SOX2* and *SOX6*), here termed as DAprog-1 and DAprog-2 (light and dark gray, respectively (Fig. 4H). The other two clusters, DA-1 and DA-2, were more mature populations expressing higher levels of late DA markers, including *NR4A2*, *DDC* and *TH* (Fig. 4I). Within DAprog-1, we found a proliferative signature (*CCND1*, *CENPF* and *E2F3*), together with pro-neural basic helix-loop-helix factor *ASCL1* expression (Fig. 4H-J)*.* The neural stem/progenitor transcription factor *SOX2*, the PD neuroprotective factor *ANXA1* and *SOX6*, which is known to have a crucial role in regulating the specification of DA neurons in the substantia nigra (Panman et al., 2014), were also expressed (Fig. 4J). whereas DAprog-1 shows lower levels of expression of *FOXA2* compared with DAprog-2, other floor plate markers such as *SOX2* and *SOX6* were enriched in this cluster while expressing the dopaminergic marker *NR4A2*, suggesting that this cluster is composed of immature neuron in transition from floor plate progenitors to DA neurons. DAprog-2 was characterized by the loss of proliferative markers and pro-neural factors, and acquired transcription factors regulating DA neuron specification, such as *EN1*, *NURR1* and *PBX1.*

Interestingly*, SNCG*, which encodes member of the synuclein family of proteins and the neural receptor *NETO2*, which was recently identified in a human DA neuronal dataset (La Manno et al., 2016; Fiorenzano et al., 2021), were also significantly seen in this subcluster. Pseudo-temporal analysis, which is used to identify trajectories between cell types/states, performed using the Slingshot method on the DA neurons confirmed the trajectory from immature to mature DA neurons with one single trajectory being identified originating from the DAprog-1 (Fig. S4C). These trajectories followed similar pattern of pseudo-temporal distribution found in the uncultured dataset (Fig. S1G). Temporally expressed genes included *GAP43*, *STMN2* and *PBX1* as late genes, whereas *VIM* and *IGFBP5* were expressed during transition (Fig. S4D). To detect whether similar progenitor populations are represented across the uncultured and cultured conditions, we performed a direct comparison of gene expression for selected markers across RG-1 and RG-2 (uncultured) and DA-prog1 and DA-prog2 (Fig. S4E). These two cell types shared many midbrain FP progenitors markers; however, unlike DA-prog, RG-1 showed a strong proliferative signature (*CCNB2*, *TOP2A* and *MKI67*).

Both DA-1 and DA-2 showed comparable enrichment in *TH* and *DLK1* expression (Fig. 4J). However, DA-1 displayed lower levels of *DAT* and was primarily enriched for *OTX2*, *SLC17A6* (also known as *VGLUT2*) and *POU2F2* (Fig. 4J), in line with what has recently been found in both human and mouse DA neuron scRNA-seq datasets (Tiklová et al., 2019; La Manno et al., 2016). DA-1 was also molecularly defined by the expression of *C1QL1*, which is involved in DA synapse formation, and *CXCR4*, which is required for the migration and projection orientation of A9-A10 DA neurons. In contrast, DA-2 expressed high levels of *DAT* and was also enriched in the proteins involved in DA catabolism and transport (*ALDH1A1* and *VMAT2*), and in *PITX3* together with its transcriptional co-activator LMO3 (Fig. 4J). To further characterize the cell type diversity across the dopaminergic clusters, we performed expression analysis of GABAergic (*GAD1*, *GAD2* and *SLC6A13*), glutamatergic (*GRIA1*, *GRIN1* and *SLC17A7*) and dopaminergic (*TH*) markers (Fig. S4F). Both clusters indicated a predominant expression of *TH* and low expression levels of glutamate- and GABA-associated genes. In comparison with DA-1, DA-2 shows higher levels of glutamate-related genes, as previously shown to be expressed in mouse DA neurons (Tiklová et al., 2019).

To compare the uncultured and cultured datasets, we used single-cell weighted correlation network analysis (scWGCNA, https://github.com/smorabit/hdWGCNA) focusing on the occurrence of transcriptome-wide gene co-expression modules in the dopaminergic neurons (Fig. S5A). WGCNA module detection was followed by gene ontology (GO) term enrichment analysis and revealed six co-expressed gene modules in the cultured dopaminergic neurons (Fig. S5B). Functions of the modules were defined by enrichment analysis (Fig. S5C). When compared with uncultured dopaminergic neurons, high expression of specific modules (‘green’, ‘turquoise’ and ‘blue’) was found in the most mature DA neurons (Fig. S5D and Table S2), as also shown by enrichment in the ‘synaptic transmission’ GO term in the blue module. Furthermore, the DA compartment emerging from 3D primary VM cultures showed a different composition from that in uncultured tissues, exhibiting a more mature human DA neuron molecular profile.

Additionally, a recent dataset (Kamath et al., 2022) shows the presence of human and primate specific DA-related gene expression, which further points to the need for cell-based model systems of human origin. *CALB1* and *GEM* did not appear to be expressed in a particular subset of DA neurons but were robustly expressed in both primary and cultured DA neurons with an increased expression of *GEM* after culture (Fig. 4K, *P*<0.001), indicating that this system might be useful for studying these human-specific genes. Finally, the DA neuron transcriptional profiles generated in this dataset (DA-1/DA-2) were compared with available profiles of adult human DA neurons from post-mortem specimens (*n*=72; Agarwal et al., 2020). 546 genes were differentially expressed between the two DA types (|logFC|>0.25 and adjusted *P*-value<0.05) with post-mortem neurons showing an enrichment for genes involved in synaptic transmission and cell adhesion, as well as glutamate and GABA signaling (Fig. S5E and Table S3).

### DISCUSSION

Animal models have been instrumental in understanding neurodevelopmental and neurodegenerative disorders, but their limitations in revealing crucial features of developmental, genetic and pathological mechanisms unique to humans are increasingly recognized. However, the inaccessibility of human brain tissue makes such studies extremely challenging, and PSC-based models do not capture all aspects of human development. Here, we performed extensive scRNA-seq analysis to decode VM development in human embryos from onset to peak DA genesis, established a 3D organoid-like culture system that supported DA neuron maturation, and used this system to profile functionally mature DA neurons in 3D cultures derived from the human fetal VM.

We and others have previously reported transcriptional profiling of human fetal VM tissue using Smart-seq2 (La Manno et al., 2016; Tiklová et al., 2020). In this study, we used high-throughput droplet-based seq (10x) to enable an analysis of much larger cell numbers. We obtained data from a total of 23,483 VM cells from three fetuses of gestational age 6-11 weeks PC. As expected, most of the cells at these early developmental timepoints were RG and FPP, in agreement with what was observed in a previous study (La Manno et al., 2016). However, three distinct neuronal subtypes were also detected: DA neurons, OTN neurons, which are all formed in the developing VM, and a small population of serotonergic neurons, which is known to be located in close proximity to DA neurons during development (Marklund et al., 2014). Moreover, the transcriptional analysis of human DA progenitors and neurons is provided here. However, one caveat to consider when trying to resolve cells that are close in the transcriptome space is that the number of recovered cells, sequencing methodology and sequencing depth could bias our interpretation of the molecular sub-identities. In this study, we used scRNA-seq yet captured more than 14,000 DA neurons, including neurons with a mature transcriptional profile with a mean depth of 1151 genes per cell. Thus, single nucleus RNA-seq, which is often superior for capturing neurons when isolating cells from the adult brain and is now often the preferred strategy for such studies (Ding et al., 2020), was not necessary to transcriptionally profile DA neurons and progenitors from fetal tissue and cultures thereof. This is advantageous as sn-RNAseq can be associated with reduced gene recovery and potential bias in the cell types recovered (Denisenko et al., 2020).

Given the even more limited accessibility of fetal tissue after the first trimester and the inherent problems with using such tissue, we established primary VM cultures to profile more mature human DA neurons. We first performed traditional monolayer (2D) cultures, but these proved inadequate for maintenance of TH neurons, prompting us to develop a 3D organoid-like culture system (Lancaster et al., 2013; Quadrato et al., 2017) that allowed the long-term maintenance and maturation of these dopaminergic cells. The 3D cultures were used to study the developmental trajectory of DA progenitors to functionally mature DA neurons expressing TH (the rate-limiting enzyme in DA synthesis) as well as enzymatic and transporter components of DA neurotransmission. We could also detect the expression of human- and primate-specific DA-related gene expression of *CALB1* and *GEM* (Kamath et al., 2022). The analysis revealed four major populations of human DA neurons: two were characterized by expression of pro-neural factors and markers of DA progenitors; and the remainder displayed the expression of more mature DA markers. A previous study based on sequencing fetal VM tissue using Smart-seq2 identified DA subclusters named DA0, DA1 and DA2 (La Manno et al., 2016). In line with our data, these subclusters differed based on the expression of genes such as *TH*, *PITX3*, *EN1* and *TMCC3* (DA0, DA1 and DA2), *SLC6A3*, *NETO2* and *KCNJ6* (DA1 and DA2), and *LMO3* and *ALDH1A1* (DA2). Most of these genes were also detected in our dataset but did not always segregate into the same clusters. This may be due partly to differences in the sequencing methods adopted (Smart-seq2 vs 10x) and cell numbers analyzed (122 analyzed by La Manno et al., 2016), but more likely reflects the fact that the molecular subtype identities present at early differentiation stages are refined as the DA neurons mature.

Of note, the expression of *GIRK2* (*KCNJ6*) and *CALB1*, commonly used to segregate the A9 and A10 DA neurons in the adult midbrain, was not found to be enriched in our DA population or in the datasets of La Manno et al. (2016), indicating that at this developmental stage, they had not yet segregated into specific DA subtypes (Reyes et al., 2012; McRitchie and Halliday, 1995). However, *ALDH1A1* was exclusively expressed in cluster DA2, suggesting that it may be an earlier marker for identifying the subtype specific A9 DA neurons. Indeed, one limitation of the datasets presented here is the lack of access to, and thus analysis of, fetal and postnatal stages where specific DA neuron subtypes are expected to emerge. Therefore, the transcriptional analysis of functionally mature human DA neurons has been performed in primary cultures in this study, and neither the timing of maturation nor the exact recapitulation of developmental events has fully been captured. However, consistent with previous results (Gordon et al., 2021), our study suggests that 3D cultures can retain mature neuronal profiles like those found in postnatal or adult brain. Indeed, our results show the presence of functionally active DA neurons already expressing *DAT*, *CALB1* and *GIRK2* after 30 days in 3D cultures, while the human fetal VM DA neurons have not yet reached this stage at the corresponding gestational time points. Although the second/third trimester stages and early postnatal stages may never be possible to investigate directly, future studies will be needed to analyze human DA neurons in extended long-term cultures or after transplantation and to compare the gene expression pattern with that of emerging datasets from human postmortem brain tissue.

Furthermore, single-cell resolution analysis of a higher number of VM fetal samples combined with cellular barcoding technology at different timepoints may allow DA lineage tracing. Combining the two approaches may help define the interrelationship between the DA neuron clusters, and reconstruct the temporal appearance and developmental trajectory of the individual DA cell types identified. Overall, this work lays the foundation for generating and maturing human DA neurons in 3D culture systems with the potential to be used for studying human VM development and DA neuron diversification. Comparative studies of gene expression, phenotypic identity and functional properties between fetal ventral midbrain (VM)-derived DA neurons and stem cell-derived DA neurons after transplantation have been vital in establishing differentiation protocols and advancing stem cell-derived DA neurons towards clinical use (Tiklová et al., 2020; Kikuchi et al., 2017; Grealish et al., 2014; Kirkeby et al., 2012). For refinement and more precise differentiation into different midbrain DA neuron subtypes, it is essential to continue to map the molecular diversity of DA neurons during development and adulthood. The data presented in this study provide a unique molecular characterization of the developing and functionally mature human DA neurons in a 3D culture system. Thus, this work will serve as a valuable resource for future advancements using stem cell-derived and reprogrammed human DA neurons *in vitro* and *in vivo*.

## MATERIALS AND METHODS

### Human embryonic tissue source

Human fetal tissues were collected from 6-11 weeks PC legally terminated embryos at Malmö Hospital (Malmö, Sweden) and Addenbrooke's Hospital (Cambridge, UK). Ethical approval for the use of postmortem human fetal tissue was provided by the Swedish National Board of Health and Welfare under existing guidelines, including informed consent from women seeking abortions, and by the National Research Ethics Service Committee East of England – Cambridge Central (Local Research Ethics Committee, 96/085). The gestational age of each embryo was determined by crown-to-rump length (CRL) (mm) measured at either the time of dissection when the quality of the embryo allowed for this or otherwise estimated by ultrasound measurements before abortion. The external features of the embryo were also carefully monitored to confirm that the CRL correlated with the appropriate embryonic stage. Samples from the UK were shipped overnight on ice in Hibernate media (ThermoFisher Scientific) to Sweden.

### Acutely dissociated cell preparations and culture conditions

Tissue from both Sweden and the UK was dissected in Hibernate media. A narrow sub-dissection of the human VM was performed, and the tissue was washed in phosphate-buffered saline (PBS) solution. Whole dissected VM tissues were used for scRNA-seq and immunohistochemical analysis of intact VM. When culturing in 2D and 3D, the same embryo was split across these two conditions in at least three replicates each (see Table S1 for details on sample and performed analysis). After 3 washes, the tissue was treated with Accutase (PAA Laboratories) for 20 min at 37°C degrees. After incubation, mechanical dissociation generated single-cell suspensions and the cells were plated at a density of 70,000 cells/well (36,842 cells/cm^2^) in culture media. Culture media used was formulated as follows: Neurobasal medium, 2 nM L-glutamine, 100 µg/ml penicillin/streptomycin, 20 ng/ml BDNF, 10 ng/ml GDNF, 0.2 mM AA and 1/3 B27. The culture media was supplemented with Y-27632 (10 µM) to improve cell survival on the plating day. A total of 1% minimum essential medium-non essential amino acids (MEM-NEAA) and 0.1% 2-mercaptoethanol were added to the culture media from day 14. The media was changed every 2 days. Two-dimensional (2D) cultures were performed in standard plates coated with a combination of polyornithine (15 µg/ml), fibronectin (0.5 ng/µl) and laminin (5 µg/ml). Three-dimensional (3D) cultures were generated using U-bottom-shaped ultra-low attachment 96-well plates (Corning). 2D and 3D conditions were set up using the sample individual. Droplets of Matrigel were applied to allow embedding at day 30 to sustain long-term cultures. At the time of embedding, 3D hVM cultures were transferred into ultra-low attachment 24-well plates (Corning). 3D cultured organoids used for calcium imaging were left attached on glass coverslips coated with polyornithine, fibronectin and laminin at day 90.

### 3D hVM culture preparation for GCaMP3 recordings

On day 95, when the 3D hVM cultures had attached to the glass coverslips, cells were transduced with the lentivirus MAP2-GCaMP3 in culture media overnight. The following day, a complete media change was performed. Before calcium imaging recordings, media were changed regularly every 2 days. Recordings were performed 7 days after viral infection.

### Immunocytochemistry and immunohistochemistry

The cells were fixed in 4% paraformaldehyde solution for 15 min at room temperature before staining. The cells were pre-incubated in a blocking solution containing 0.1 M PBS with potassium (KPBS), 0.1% Triton and 5% serum (of secondary antibody host species) for 1-3 h before the primary antibody solution was added.

3D cultures were fixed in 4% paraformaldehyde overnight at room temperature and cryoprotected in 30% sucrose before frozen in Tissue-Tek OCT (Sakura FineTek). Sectioning of the frozen 3D samples was performed using a cryostat supplier, with slices of 200 µm.

The cells were incubated with the primary antibodies overnight at 4°C and the following day they were washed with KPBS before adding the secondary antibody solution containing fluorophore-conjugated antibodies (1:200, Jackson ImmunoResearch Laboratories) and DAPI (1:500). The cells were incubated with the secondary antibodies for 2 h at room temperature and finally washed with KPBS. Primary antibodies used were rabbit anti-tyrosine hydroxylase (TH) (1:1000, Pel-Freeze Biological, P40101-150), goat anti-FOXA2 (1:500, R&D Systems, HAF019), rabbit anti-OLIG2 (1:500, Neuromics, RA25081), mouse anti-SOX2 (1:50, R&D Systems, MAB2018), mouse anti-nestin (1:500, BD Bioscience, 611658), mouse anti-microtubule-associated protein 2 (Map2, 1:250, Sigma-Aldrich, 4133), rabbit anti-glial fibrillary acidic protein (GFAP, 1:1000, DAKO, Z0334), rabbit anti DDC (1:1000, Millipore, AB1569), rabbit anti-DAT (1:200, Santa Cruz Biotechnology, sc-14002), goat anti-OTX2 (1:2000, R&D Systems, AF1979), rabbit anti-CALB (1:500, Swant, CB-38a), goat anti-GIRK2 (1:200, abcam, ab65096), rabbit anti-ALDH1A1 (1:200, Abcam, ab52492).

### scRNA-seq analysis

Cell suspensions were loaded into a 10x Genomics Chromium Single Cell System (10x Genomics) and libraries were generated using version 3 chemistry according to the manufacturer's instructions. Libraries were sequenced on an Illumina NextSeq500 (400 million reads) using the recommended read length. Sequencing data were first pre-processed through the Cell Ranger pipeline (10x Genomics, Cellranger count v2) with default parameters, aligned to GrCH38 (v3.1.0) and resulting matrix files were used for subsequent bioinformatic analysis. Seurat (version 3.1.1 and R version 3.6.1) was used for downstream analysis. Batch effects were removed using the Harmony algorithm (1.0), treating individual 10x runs as a batch. Cells with at least 200 detected genes were retained and the data were normalized to transcript copies per 10,000, and log-normalized to reduce sequencing depth variability. Average number of genes expressed for uncultured dataset was 1102 genes/cell; for DA neurons it was 1151 genes/cell. For visualization and clustering, manifolds were calculated using UMAP methods (RunUMAP, Seurat) using 20 precomputed principal components and the shared nearest neighbor algorithm modularity optimization-based clustering algorithm (FindClusters, Seurat) with a resolution of 0.2. Analysis of the uncultured human fetal tissue was initially performed grouping all the samples to provide a more accurate cell type assignment. Cell type assignment was based on the previously published classification of La Manno et al. (2016) using singleR methodology. This step was followed by analyzing each sample separately and a quantification of the contribution of each cell cluster per sample was performed.

For analysis of DA neurons in 3D cultures, the selection of DA sub-clusters was performed based on cell-type assignment referencing the dataset published by La Manno et al. (2016) combined with label transfer from the uncultured dataset. Silhouette analysis was performed to validate clustering classification and the highest silhouette score indicated that cells being matched with one cluster are poorly matched with neighboring clusters. Single-cell weighted correlation network analysis (scWGCNA, https://github.com/smorabit/hdWGCNA) was performed to determine the occurrence of transcriptome-wide gene co-expression modules in the dopaminergic neurons with parameters fraction=0.05, k=25 and 15 pre-computed UMAP dimensions. WGCNA module detection was followed by gene ontology [GO, RunEnrichr (Seurat)] term enrichment analysis to reveal the co-expressed gene modules. Enrichment analysis defined the function of the modules. Identification of differentially expressed genes between clusters was carried out using the default Wilcoxon rank sum test (Seurat). Gene ontology overrepresentation analysis was performed using the enrichGO function in the clusterProfiler package (3.13) using MSigDB as the database. Differentiation trajectories and pseudotemporal ordering of cells was carried out using the DiffusionMap function (destiny, version 3.4) with 50 pre-computed principal components using a sigma of 31.60. For pseudotemporal ordering of DA neurons, Slingshot was applied (version 1.80). Comparison of postmortem data was made using a previously published dataset (Agarwal et al., 2020).

### iDISCO

3D fetal cultures were fixed in 2% paraformaldehyde overnight at 4°C followed by permeabilization in 0.2% Triton X-100/20% DMSO. After 2 h, organoids were incubated overnight in 0.1% Triton X-100, 0.1% Tween20, 0.1% C_24_H_39_NA0_4_, 0.1% NP40 and 20% DMSO at 37°C. Cultures were incubated with primary antibodies for 2 days at 37°C followed by a 2-day incubation with secondary antibodies, embedded in 1% agarose and dehydrated in an ascending concentration series of methanol and dichloromethane, as previously described (Renier et al., 2014). Fetal VM and organoids were imaged on a Ultra microscope II Light-Sheet Microscope (LaVision Biotec) equipped with a sCMOS camera (Andor Neo, model 5.5-CL3) and 4× objective lens (LaVision LVMI-Floor 4×/0.3 WD6) or 12× objective lens (NA 0.53 MI PLAN DC57 WD10 OI) equipped with a 6 mm working distance dipping cap. We used two laser configurations with the following emission filters: 525/50 for TH staining on organoid and 680/30 for Alexa Flour-647 on fetal embryo. Stacks were acquired with capturing software ImspectorPro64 (LaVision Biotec) using 5 µm *z*-steps. They were imaged in a chamber filled with DBE. These image stacks were assembled to visualize the brain in 3D with Arivis Vision 4D 3.0. Rendered movies were assembled in Final Cut Pro 10.4.3 (Apple).

### Microscopy

Fluorescent images were captured using a Leica DMI6000B wide-field microscope. Image acquisition software was Leica LAS X and images were processed using Adobe Photoshop CC 2018. Any adjustments were applied equally across the entire image and without the loss of any information. Immunohistochemical stainings were analyzed using a Leica confocal microscope with 20×/0.70 IMM and HP PL APO CS2 63×/1.20 water objectives. Double staining was confirmed by conduction of high-magnified confocal *z*-stacks. All figures were assembled using Canvas software.

### Calcium imaging of MAP2-GCamP3-labeled neurons

Calcium imaging was performed at day 100 of 3D hVM cultures containing the MAP2-GCamP3 reporter. Cell culture media were replaced with 100 µl baseline buffer containing 1.2×10^−3^ M MgCl_2_, 2×10^−3^ M CaCl_2_, 150×10^−3^ M NaCl, 5×10^−3^ M KCl, 5×10^−3^ M glucose and 10×10^−3^ M HEPES. Imaging was performed on an inverted Ti2 microscope (Nikon) equipped with a CSU–W1 spinning disc system (Yokogawa), a sCMOS camera (Teledyne Photometrics) and a 20× objective lens. An environment control chamber was used to maintain the temperature at 37°C and CO_2_ level at 5% during imaging. Exposure time was set to 30 ms or 100 ms, depending on the dynamics of calcium transients. Spontaneous activity was recorded from three different 3D fetal structures from different embryos. Images were analyzed in ImageJ (NIH) and plotted in QtiPlot.

### Whole-cell patch-clamp recordings

Cells from the 2D condition model were cultured on glass coverslips and transferred to a recording chamber with a constant flow of Krebs solution gassed with 95% O_2_/5% CO_2_ at room temperature. The composition of the standard solution was (in mM): 119 NaCl, 2.5 KCl, 1.3 MgSO_4_, 2.5 CaCl_2_, 25 glucose and 26 NaHCO_3_. 3D cultured hVMs were maintained in ultra-low attachment plates. On recording day, 3D hVMs were transferred to a recording chamber with Krebs solution gassed with 95% O_2_/5% CO_2_ at room temperature. For recordings, a Multiclamp 700B amplifier (Molecular Devices) was used together with borosilicate glass pipettes (3-7 MOhm) filled with the following intracellular solution (in mM): 122.5 C6H11KO7, 12.5 KCl, 0.2 EGTA, 10 HEPES, 2 MgATP, 0.3 Na_3_GTP and 8 NaCl adjusted to pH 7.3 with KOH as previously described (Pfisterer et al., 2011). Data acquisition was performed with pClamp 10.2 (Molecular Devices); current was filtered at 0.1 kHz and digitized at 2 kHz. Cells with neuronal morphology and round cell body were selected for recordings. RMPs were monitored immediately after breaking-in in current-clamp mode. Thereafter, cells were kept at a membrane potential of −45 mV to −70 mV, and 500 ms currents were injected from −50 pA to +100 pA with 10 pA increments to induce action potentials. For inward sodium and delayed rectifying potassium current measurements, cells were clamped at −70 mV and voltage-depolarizing steps were delivered for 100 ms at 10 mV increments. Spontaneous APs were recorded in current-clamp mode at RMPs. Postsynaptic activity was recorded in voltage-clamp mode at RMPs.

### Statistical analysis

All the quantitative analysis in 2D and 3D experiments was carried out by comparing cultures derived from the same individual. All data are expressed as mean±s.e.m. A Shapiro–Wilk normality test was used to assess the normality of the distribution, and parametric or nonparametric tests were performed accordingly. Statistical analyses were conducted using GraphPad Prism 8.0.

## Supplementary Material

10.1242/develop.200504_sup1 Supplementary information

## References

[DEV200504C1] Agarwal, D., Sandor, C., Volpato, V., Caffrey, T. M., Monzón-Sandoval, J., Bowden, R., Alegre-Abarrategui, J., Wade-Martins, R. and Webber, C. (2020). A single-cell atlas of the human substantia nigra reveals cell-specific pathways associated with neurological disorders. *Nat. Commun.* 11, 4183. 10.1038/s41467-020-17876-0 32826893 PMC7442652

[DEV200504C2] Ambasudhan, R., Talantova, M., Coleman, R., Yuan, X., Zhu, S., Lipton, S. A. and Ding, S. (2011). Direct reprogramming of adult human fibroblasts to functional neurons under defined conditions. *Cell Stem Cell* 9, 113-118. 10.1016/j.stem.2011.07.002 21802386 PMC4567246

[DEV200504C3] Anderegg, A., Poulin, J.-F. and Awatramani, R. (2015). Molecular heterogeneity of midbrain dopaminergic neurons--Moving toward single cell resolution. *FEBS Lett.* 589 (24 Pt A), 3714-3726. 10.1016/j.febslet.2015.10.022 26505674 PMC4679573

[DEV200504C4] Björklund, A. and Dunnett, S. B. (2007). Dopamine neuron systems in the brain: an update. *Trends Neurosci.* 30, 194-202. 10.1016/j.tins.2007.03.006 17408759

[DEV200504C5] Cao, J., Packer, J. S., Ramani, V., Cusanovich, D. A., Huynh, C., Daza, R., Qiu, X., Lee, C., Furlan, S. N., Steemers, F. J. et al. (2017). Comprehensive single-cell transcriptional profiling of a multicellular organism. *Science* 357, 661-667. 10.1126/science.aam8940 28818938 PMC5894354

[DEV200504C6] Chergui, K., Charléty, P. J., Akaoka, H., Saunier, C. F., Brunet, J.-L., Buda, M., Svensson, T. H. and Chouvet, G. (1993). Tonic activation of NMDA receptors causes spontaneous burst discharge of rat midbrain dopamine neurons in vivo. *Eur. J. Neurosci.* 5, 137-144. 10.1111/j.1460-9568.1993.tb00479.x 8261095

[DEV200504C7] Cooper, O., Seo, H., Andrabi, S., Guardia-Laguarta, C., Graziotto, J., Sundberg, M., McLean, J. R., Carrillo-Reid, L., Xie, Z., Osborn, T. et al. (2012). Pharmacological rescue of mitochondrial deficits in iPSC-derived neural cells from patients with familial Parkinson's disease. *Sci. Transl. Med.* 4, 141ra90. 10.1126/scitranslmed.3003985 PMC3462009 22764206

[DEV200504C8] Denisenko, E., Guo, B. B., Jones, M. G., Hou, R., de Kock, L., Lassmann, T., Poppe, D., Clément, O., Simmons, R. K., Lister, R. et al. (2020). Systematic assessment of tissue dissociation and storage biases in single-cell and single-nucleus RNA-seq workflows. *Genome Biol.* 21, 130. 10.1186/s13059-020-02048-6 32487174 PMC7265231

[DEV200504C9] Ding, J., Adiconis, X., Simmons, S. K., Kowalczyk, M. S., Hession, C. C., Marjanovic, N. D., Hughes, T. K., Wadsworth, M. H., Burks, T., Nguyen, L. T. et al. (2020). Systematic comparison of single-cell and single-nucleus RNA-sequencing methods. *Nat. Biotechnol.* 38, 737-746. 10.1038/s41587-020-0465-8 32341560 PMC7289686

[DEV200504C10] Doi, D., Magotani, H., Kikuchi, T., Ikeda, M., Hiramatsu, S., Yoshida, K., Amano, N., Nomura, M., Umekage, M., Morizane, A. et al. (2020). Pre-clinical study of induced pluripotent stem cell-derived dopaminergic progenitor cells for Parkinson's disease. *Nat. Commun.* 11, 3369. 10.1038/s41467-020-17165-w 32632153 PMC7338530

[DEV200504C11] Dreyer, J. K., Herrik, K. F., Berg, R. W. and Hounsgaard, J. D. (2010). Influence of phasic and tonic dopamine release on receptor activation. *J. Neurosci.* 30, 14273-14283. 10.1523/JNEUROSCI.1894-10.2010 20962248 PMC6634758

[DEV200504C12] Fiorenzano, A., Sozzi, E., Birtele, M., Kajtez, J., Giacomoni, J., Nilsson, F., Bruzelius, A., Sharma, Y., Zhang, Y., Mattsson, B. et al. (2021). Single-cell transcriptomics captures features of human midbrain development and dopamine neuron diversity in brain organoids. *Nat. Commun.* 12, 7302. 10.1038/s41467-021-27464-5 34911939 PMC8674361

[DEV200504C13] Gordon, A., Yoon, S.-J., Tran, S. S., Makinson, C. D., Park, J. Y., Andersen, J., Valencia, A. M., Horvath, S., Xiao, X., Huguenard, J. R. et al. (2021). Long-term maturation of human cortical organoids matches key early postnatal transitions. *Nat. Neurosci.* 24, 331-342. 10.1038/s41593-021-00802-y 33619405 PMC8109149

[DEV200504C14] Grace, A. A. (2016). Dysregulation of the dopamine system in the pathophysiology of schizophrenia and depression. *Nat. Rev. Neurosci.* 17, 524-532. 10.1038/nrn.2016.57 27256556 PMC5166560

[DEV200504C15] Grealish, S., Diguet, E., Kirkeby, A., Mattsson, B., Heuer, A., Bramoulle, Y., Van Camp, N., Perrier, A. L., Hantraye, P., Björklund, A. et al. (2014). Human ESC-derived dopamine neurons show similar preclinical efficacy and potency to fetal neurons when grafted in a rat model of Parkinson's disease. *Cell Stem Cell* 15, 653-665. 10.1016/j.stem.2014.09.017 25517469 PMC4232736

[DEV200504C16] Haghverdi, L., Büttner, M., Wolf, F. A., Buettner, F. and Theis, F. J. (2016). Diffusion pseudotime robustly reconstructs lineage branching. *Nat. Methods* 13, 845-848. 10.1038/nmeth.3971 27571553

[DEV200504C17] Hook, P. W., McClymont, S. A., Cannon, G. H., Law, W. D., Morton, A. J., Goff, L. A. and McCallion, A. S. (2018). Single-cell RNA-seq of mouse dopaminergic neurons informs candidate gene selection for sporadic Parkinson disease. *Am. J. Hum. Genet.* 102, 427-446. 10.1016/j.ajhg.2018.02.001 29499164 PMC5985341

[DEV200504C18] Kamath, T., Abdulraouf, A., Burris, S. J., Langlieb, J., Gazestani, V., Nadaf, N. M., Balderrama, K., Vanderburg, C. and Macosko, E. Z. (2022). Single-cell genomic profiling of human dopamine neurons identifies a population that selectively degenerates in Parkinson's disease. *Nat. Neurosci.* 25, 588-595. 10.1038/s41593-022-01061-1 35513515 PMC9076534

[DEV200504C19] Ke, M., Chong, C.-M., Zeng, H., Huang, M., Huang, Z., Zhang, K., Cen, X., Lu, J.-H., Yao, X., Qin, D. et al. (2020). Azoramide protects iPSC-derived dopaminergic neurons with PLA2G6 D331Y mutation through restoring ER function and CREB signaling. *Cell Death Dis.* 11, 130. 10.1038/s41419-020-2312-8 32071291 PMC7028918

[DEV200504C20] Kee, N., Volakakis, N., Kirkeby, A., Dahl, L., Storvall, H., Nolbrant, S., Lahti, L., Björklund, Å. K., Gillberg, L., Joodmardi, E. et al. (2017). Single-cell analysis reveals a close relationship between differentiating dopamine and subthalamic nucleus neuronal lineages. *Cell Stem Cell* 20, 29-40 10.1016/j.stem.2016.10.003 28094018

[DEV200504C21] Kikuchi, T., Morizane, A., Doi, D., Magotani, H., Onoe, H., Hayashi, T., Mizuma, H., Takara, S., Takahashi, R., Inoue, H. et al. (2017). Human iPS cell-derived dopaminergic neurons function in a primate Parkinson's disease model. *Nature* 548, 592-596. 10.1038/nature23664 28858313

[DEV200504C22] Kirkeby, A., Grealish, S., Wolf, D. A., Nelander, J., Wood, J., Lundblad, M., Lindvall, O. and Parmar, M. (2012). Generation of regionally specified neural progenitors and functional neurons from human embryonic stem cells under defined conditions. *Cell Rep.* 1, 703-714. 10.1016/j.celrep.2012.04.009 22813745

[DEV200504C23] Kirkeby, A., Nolbrant, S., Tiklova, K., Heuer, A., Kee, N., Cardoso, T., Ottosson, D. R., Lelos, M. J., Rifes, P., Dunnett, S. B. et al. (2017). Predictive markers guide differentiation to improve graft outcome in clinical translation of hESC-based therapy for Parkinson's disease. *Cell Stem Cell* 20, 135-148. 10.1016/j.stem.2016.09.004 28094017 PMC5222722

[DEV200504C24] Kouroupi, G., Taoufik, E., Vlachos, I. S., Tsioras, K., Antoniou, N., Papastefanaki, F., Chroni-Tzartou, D., Wrasidlo, W., Bohl, D., Stellas, D. et al. (2017). Defective synaptic connectivity and axonal neuropathology in a human iPSC-based model of familial Parkinson's disease. *Proc. Natl. Acad. Sci. USA* 114, E3679-E3688. 10.1073/pnas.1617259114 28416701 PMC5422768

[DEV200504C25] La Manno, G., Gyllborg, D., Codeluppi, S., Nishimura, K., Salto, C., Zeisel, A., Borm, L. E., Stott, S. R. W., Toledo, E. M., Villaescusa, J. C. et al. (2016). Molecular diversity of midbrain development in mouse, human, and stem cells. *Cell* 167, 566-580.e19. 10.1016/j.cell.2016.09.027 27716510 PMC5055122

[DEV200504C26] Lancaster, M. A., Renner, M., Martin, C.-A., Wenzel, D., Bicknell, L. S., Hurles, M. E., Homfray, T., Penninger, J. M., Jackson, A. P. and Knoblich, J. A. (2013). Cerebral organoids model human brain development and microcephaly. *Nature* 501, 373-379. 10.1038/nature12517 23995685 PMC3817409

[DEV200504C27] Marklund, U., Alekseenko, Z., Andersson, E., Falci, S., Westgren, M., Perlmann, T., Graham, A., Sundström, E. and Ericson, J. (2014). Detailed expression analysis of regulatory genes in the early developing human neural tube. *Stem Cells Dev.* 23, 5-15. 10.1089/scd.2013.0309 24007338 PMC3870486

[DEV200504C28] McRitchie, D. A. and Halliday, G. M. (1995). Calbindin D28k-containing neurons are restricted to the medial substantia nigra in humans. *Neuroscience* 65, 87-91. 10.1016/0306-4522(94)00483-L 7538646

[DEV200504C29] Nedergaard, S. (1999). Regulation of action potential size and excitability in substantia nigra compacta neurons: sensitivity to 4-aminopyridine. *J. Neurophysiol.* 82, 2903-2913. 10.1152/jn.1999.82.6.2903 10601428

[DEV200504C30] Nedergaard, S. (2004). A Ca2+-independent slow afterhyperpolarization in substantia nigra compacta neurons. *Neuroscience* 125, 841-852. 10.1016/j.neuroscience.2004.02.030 15120845

[DEV200504C31] Ono, Y., Nakatani, T., Sakamoto, Y., Mizuhara, E., Minaki, Y., Kumai, M., Hamaguchi, A., Nishimura, M., Inoue, Y., Hayashi, H. et al. (2007). Differences in neurogenic potential in floor plate cells along an anteroposterior location: midbrain dopaminergic neurons originate from mesencephalic floor plate cells. *Development* 134, 3213-3225. 10.1242/dev.02879 17670789

[DEV200504C32] Panman, L., Papathanou, M., Laguna, A., Oosterveen, T., Volakakis, N., Acampora, D., Kurtsdotter, I., Yoshitake, T., Kehr, J., Joodmardi, E. et al. (2014). Sox6 and Otx2 control the specification of substantia nigra and ventral tegmental area dopamine neurons. *Cell Rep.* 8, 1018-1025. 10.1016/j.celrep.2014.07.016 25127144

[DEV200504C33] Pfisterer, U., Kirkeby, A., Torper, O., Wood, J., Nelander, J., Dufour, A., Björklund, A., Lindvall, O., Jakobsson, J. and Parmar, M. (2011). Direct conversion of human fibroblasts to dopaminergic neurons. *Proc. Natl. Acad. Sci. USA* 108, 10343-10348. 10.1073/pnas.1105135108 21646515 PMC3121829

[DEV200504C34] Piao, J., Zabierowski, S., Dubose, B. N., Hill, E. J., Navare, M., Claros, N., Rosen, S., Ramnarine, K., Horn, C., Fredrickson, C. et al. (2021). Preclinical efficacy and safety of a human embryonic stem cell-derived midbrain dopamine progenitor product, MSK-DA01. *Cell Stem Cell* 28, 217-229.e7 10.1016/j.stem.2021.01.004 33545080 PMC7903922

[DEV200504C35] Poulin, J.-F., Zou, J., Drouin-Ouellet, J., Kim, K.-Y. A., Cicchetti, F. and Awatramani, R. B. (2014). Defining midbrain dopaminergic neuron diversity by single-cell gene expression profiling. *Cell Rep.* 9, 930-943. 10.1016/j.celrep.2014.10.008 25437550 PMC4251558

[DEV200504C36] Quadrato, G., Nguyen, T., Macosko, E. Z., Sherwood, J. L., Yang, S. M., Berger, D. R., Maria, N., Scholvin, J., Goldman, M., Kinney, J. P. et al. (2017). Cell diversity and network dynamics in photosensitive human brain organoids. *Nature* 545, 48-53. 10.1038/nature22047 28445462 PMC5659341

[DEV200504C37] Reinhardt, P., Schmid, B., Burbulla, L. F., Schöndorf, D. C., Wagner, L., Glatza, M., Höing, S., Hargus, G., Heck, S. A., Dhingra, A. et al. (2013). Genetic correction of a LRRK2 mutation in human iPSCs links parkinsonian neurodegeneration to ERK-dependent changes in gene expression. *Cell Stem Cell* 12, 354-367. 10.1016/j.stem.2013.01.008 23472874

[DEV200504C38] Renier, N., Wu, Z., Simon, D. J., Yang, J., Ariel, P. and Tessier-Lavigne, M. (2014). iDISCO: a simple, rapid method to immunolabel large tissue samples for volume imaging. *Cell* 159, 896-910. 10.1016/j.cell.2014.10.010 25417164

[DEV200504C39] Reyes, S., Fu, Y., Double, K., Thompson, L., Kirik, D., Paxinos, G. and Halliday, G. M. (2012). GIRK2 expression in dopamine neurons of the substantia nigra and ventral tegmental area. *J. Comp. Neurol.* 520, 2591-2607. 10.1002/cne.23051 22252428

[DEV200504C40] Roeper, J. (2013). Dissecting the diversity of midbrain dopamine neurons. *Trends Neurosci.* 36, 336-342. 10.1016/j.tins.2013.03.003 23582338

[DEV200504C41] Rosenberg, A. B., Roco, C. M., Muscat, R. A., Kuchina, A., Sample, P., Yao, Z., Graybuck, L. T., Peeler, D. J., Mukherjee, S., Chen, W. et al. (2018). Single-cell profiling of the developing mouse brain and spinal cord with split-pool barcoding. *Science* 360, 176-182. 10.1126/science.aam8999 29545511 PMC7643870

[DEV200504C42] Saunders, A., Macosko, E. Z., Wysoker, A., Goldman, M., Krienen, F. M., de Rivera, H., Bien, E., Baum, M., Bortolin, L., Wang, S. et al. (2018). Molecular diversity and specializations among the cells of the adult mouse brain. *Cell* 174, 1015-1030.e16. 10.1016/j.cell.2018.07.028 30096299 PMC6447408

[DEV200504C43] Tiklová, K., Björklund, Å. K., Lahti, L., Fiorenzano, A., Nolbrant, S., Gillberg, L., Volakakis, N., Yokota, C., Hilscher, M. M., Hauling, T. et al. (2019). Single-cell RNA sequencing reveals midbrain dopamine neuron diversity emerging during mouse brain development. *Nat. Commun.* 10, 581 10.1038/s41467-019-08453-1 30718509 PMC6362095

[DEV200504C44] Tiklová, K., Nolbrant, S., Fiorenzano, A., Björklund, Å. K., Sharma, Y., Heuer, A., Gillberg, L., Hoban, D. B., Cardoso, T., Adler, A. F. et al. (2020). Single cell transcriptomics identifies stem cell-derived graft composition in a model of Parkinson's disease. *Nat. Commun.* 11, 2434. 10.1038/s41467-020-16225-5 32415072 PMC7229159

