# Supplementary material for: Single-cell transcriptional and functional analysis of dopaminergic neurons in organoid-like cultures derived from human fetal midbrain
Source: Development. 2022 Dec 8;149(23):dev200504. doi: 10.1242/dev.200504 (PMC10114107; doi:10.1242/dev.200504)
Supplement: Supplementary information [file develop-149-200504-s1.pdf]

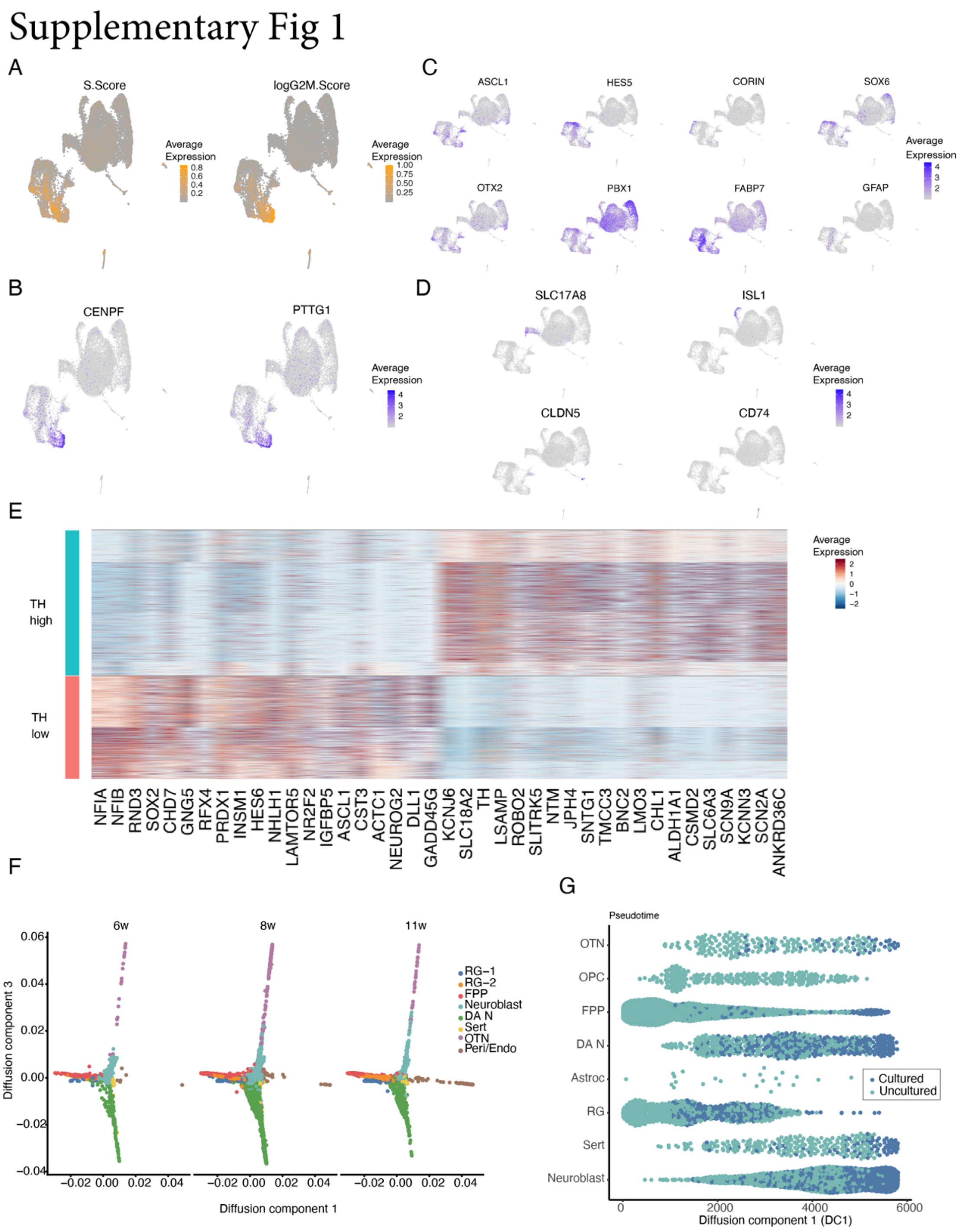

Fig. S1. Characterization of human fetal Midbrain. Related to Figure 1.

A, S.Sore and logG2M analysis. Gray-yellow colors indicate expression level. **B,** Feature plots for *CENPF* and *PTTG1* marking proliferative cells. Gray-purple colors indicate expression level. **C,** Feature plots for *ASCL1*, *HES5*, *CORIN*, *SOX6, OTX2, PBX1, FABP7* and *GFAP*. Gray-purple

Development • Supplementary information

colors indicate expression level. **D,** Feature plots for *SLC17A8, ISL1, CLDN5* and *CD74.* Gray-purple colors indicate expression level. **E,** Heatmap for TH High and TH Low groups showing expression of selected markers. Blue-red colors indicate expression level. **F,** Diffusion map for each human fetal tissue of diffusion component 1 and 3 reconstructing post-mitotic cell maturation. Cells were colored according to cell type. G, Pseudotemporal ordering of emerging cell types in uncultured and cultured samples according to DC1.

Development • Supplementary information

Suppl Fig 2

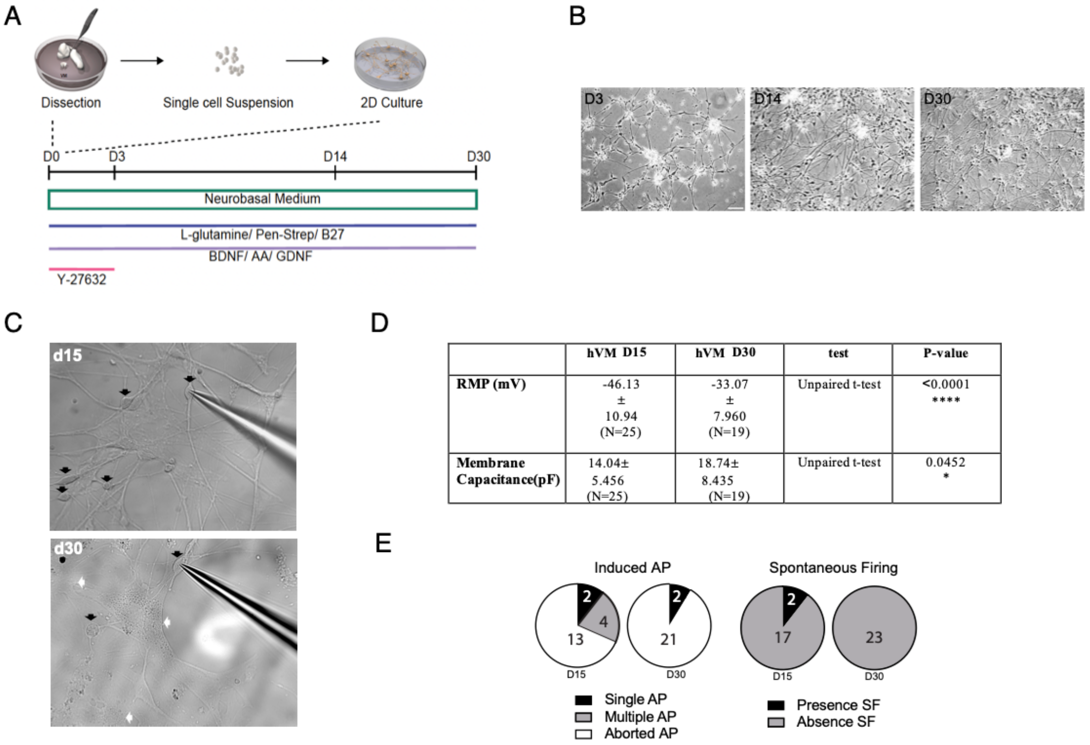

Fig. S2. Characterization of 2D cultures of hVM. Related to Figure 2.

A, Schematic overview of 2D hVM cultures. **B,** Brightfield images of d3, d14 and d30 2D hVM cultures. **C,** Bright field images representing whole-cell patch-clamp recordings at d15 and d30. Black arrows show the higher presence of neuronal like cells at d15 compared at d30. Presence of non-neuronal cell morphologies, white arrows, was easily detect at d30 but not at d15. **D,** Table representing passive properties of the membrane from whole-cell patch-clamp recordings. Values of RMP and membrane capacitance reported as mean and standard deviation. **E,** Proportion of cells firing induced action potentials and spontaneous firing at d15 and d30. Scale bars, 100µM.

Development • Supplementary information

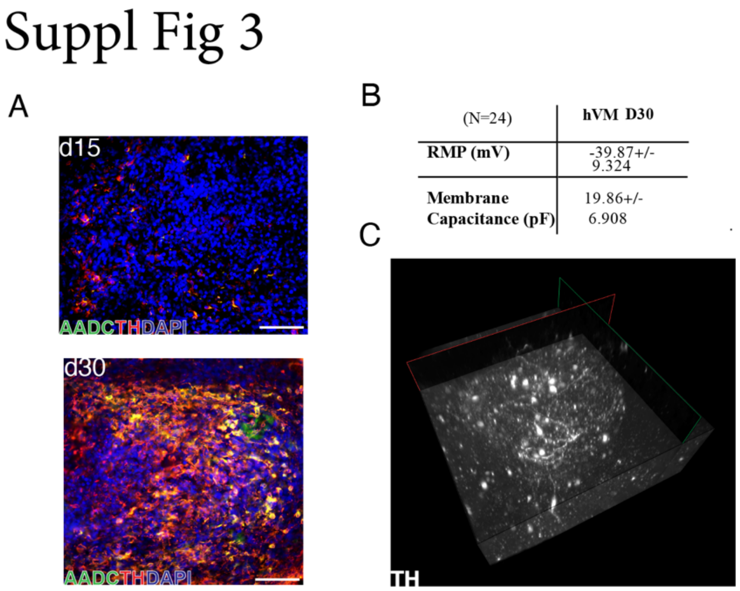

Fig. S3. Functional characterization of 3D hVM cultures. Related to Figure 3.

A, Immunohistochemical images displaying AADC, TH, DAPI positive cells at d15 and d30. Scale bar, 50 µM. **B**, Table indicating RMP and membrane capacitance from whole-cell patch-clamp recordings performed at d30. Values indicate mean and standard deviation. **C,** reconstruction from iDISCO video highlighting TH^+ neurons. Scale bars, 50 µM.

Development • Supplementary information

Suppl Fig 4

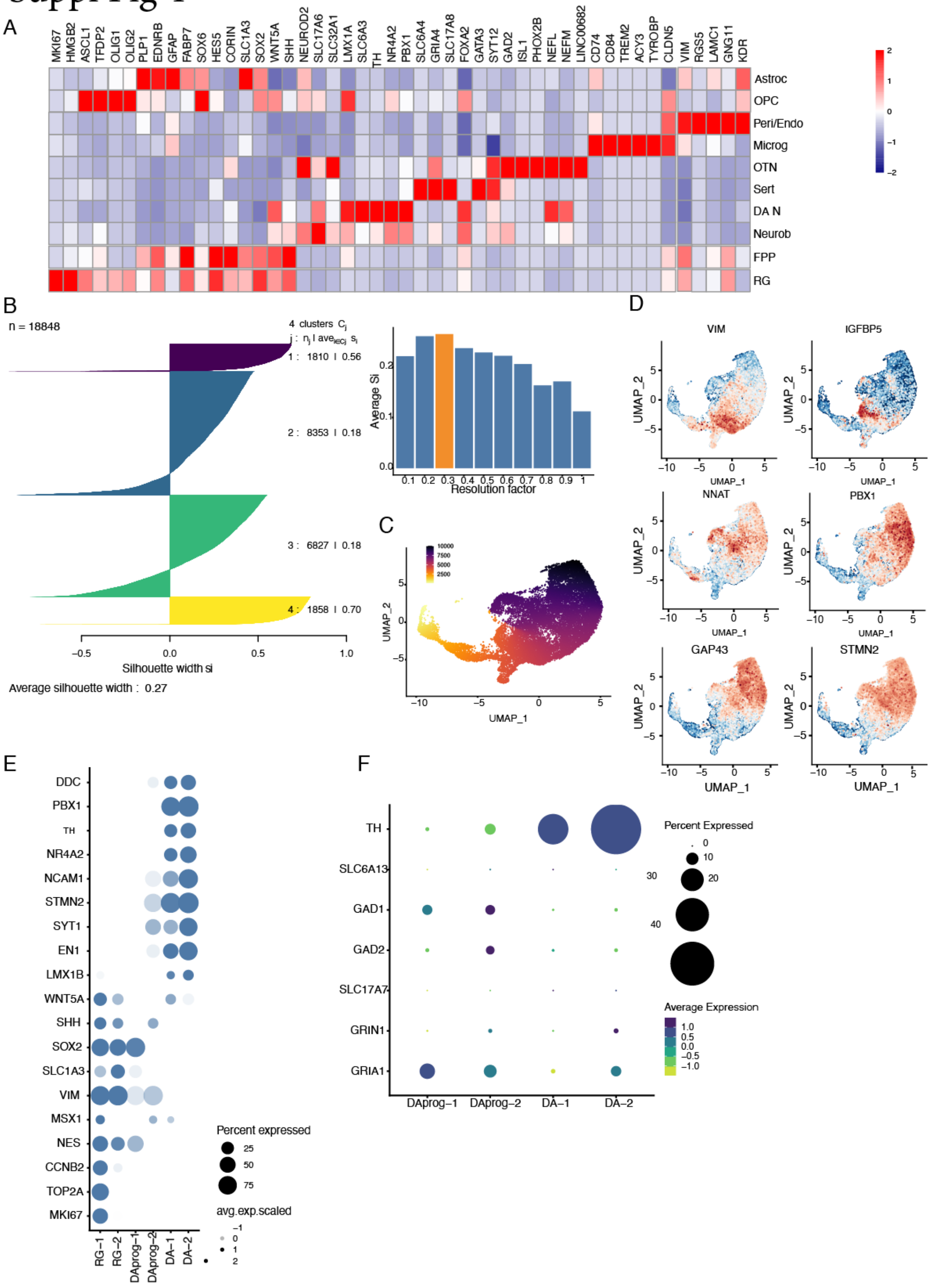

Development • Supplementary information

Fig S4. Transcriptional profile of 2D and 3D human fetal cultures. Related to Figure 4.
A, Heatmap for Astroc, OPC, Peri/endo, Microg, OTN, Sert, DA N, Neurob, FPP, and RG groups showing expression of selected markers of 2D hVM cultures. Blue-red colors indicate expression level. **B,** Silhouette analysis validating the clustering of dopaminergic cells into four groups. The highest silhouette score indicates that cells are being matched with one cluster and they are poorly matching with neighboring clusters. **C,** Pseudotime coloring of maturing dopamine neurons as identified by Slingshot. **D,** Top genes identified by modelling relationships between gene expression and pseudotime using general additive models. Cells are colored by expression level. **E**, Dot-plot showing expression of selected markers across RG-1 and RG-2 (uncultured) and DA-prog1 and DA-prog2**. F,** Expression analysis of GABAergic (GAD1-2, SLC6A13), glutamatergic (GRIA1, GRIN1, SLC17A7) and dopaminergic (TH) markers across dopaminergic clusters.

Development • Supplementary information

Suppl Fig 5

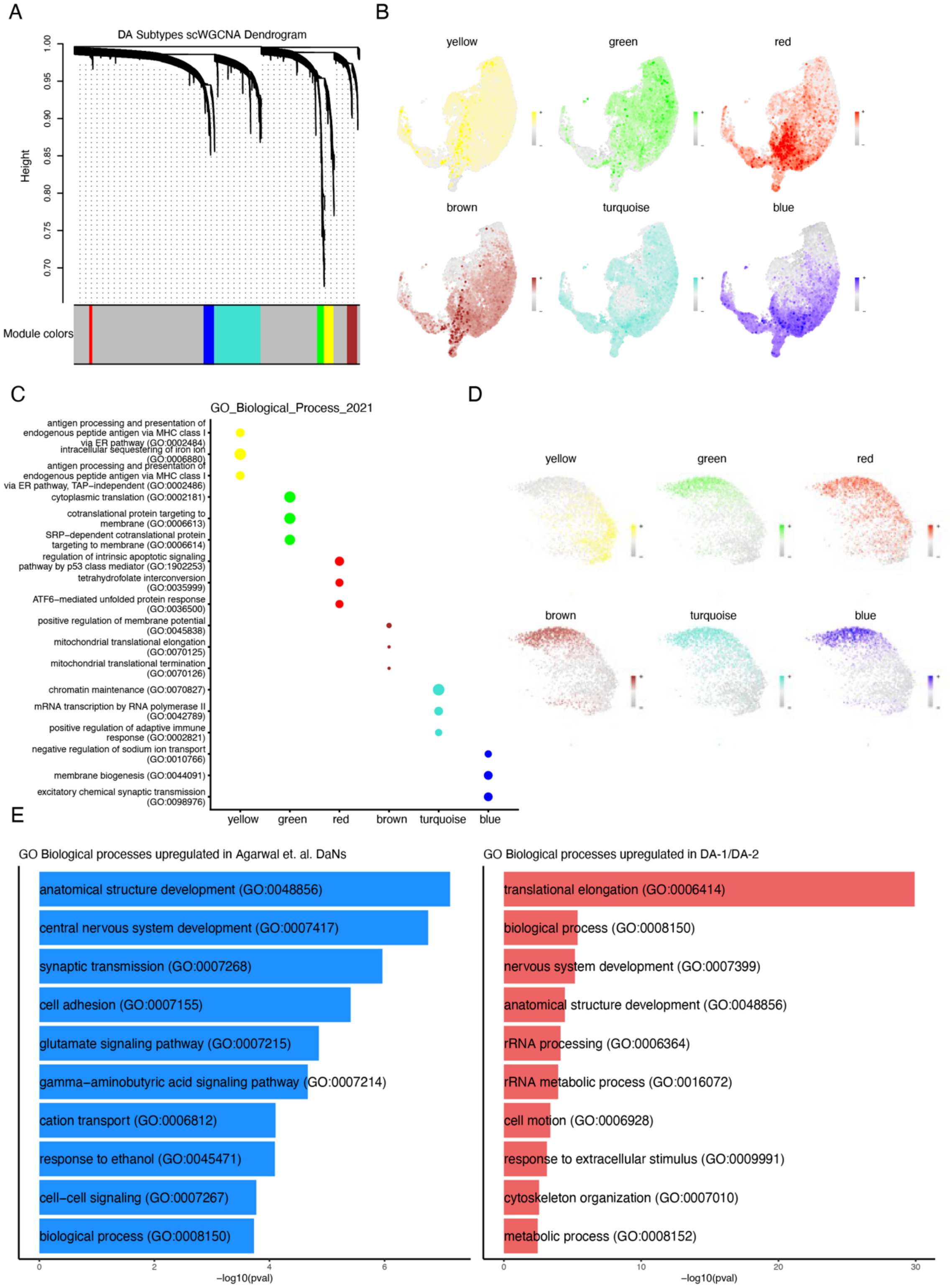

Development • Supplementary information

Fig. S5. Transcriptional profile of 2D and 3D human fetal cultures. Related to Figure 4.

A, Single-cell weighted correlation network analysis (scWGCNA) focusing on the occurrence of transcriptome-wide gene co-expression modules in the dopaminergic neurons. **B,** WGCNA module detection followed by gene ontology (GO) term enrichment analysis revealed six co-expressed gene modules in the cultured dopaminergic neurons. **C,** Function of the modules have been defined by enrichment analysis. **D,** High expression modules were found in the most mature DA neurons (Supplementary Fig. 4I). **E,** Differentially expressed gene list between dopaminergic neurons from this study and postmortem human tissue.

Table S1. Details on human fetal embryo and performed analyses.

Click here to download Table S1

Table S2. scWGCNA analysis of uncultured and cultured datasets.

Click here to download Table S2

Table S3. DEGs across human fetal dataset and adult postmortem dataset.

Click here to download Table S3

Development • Supplementary information

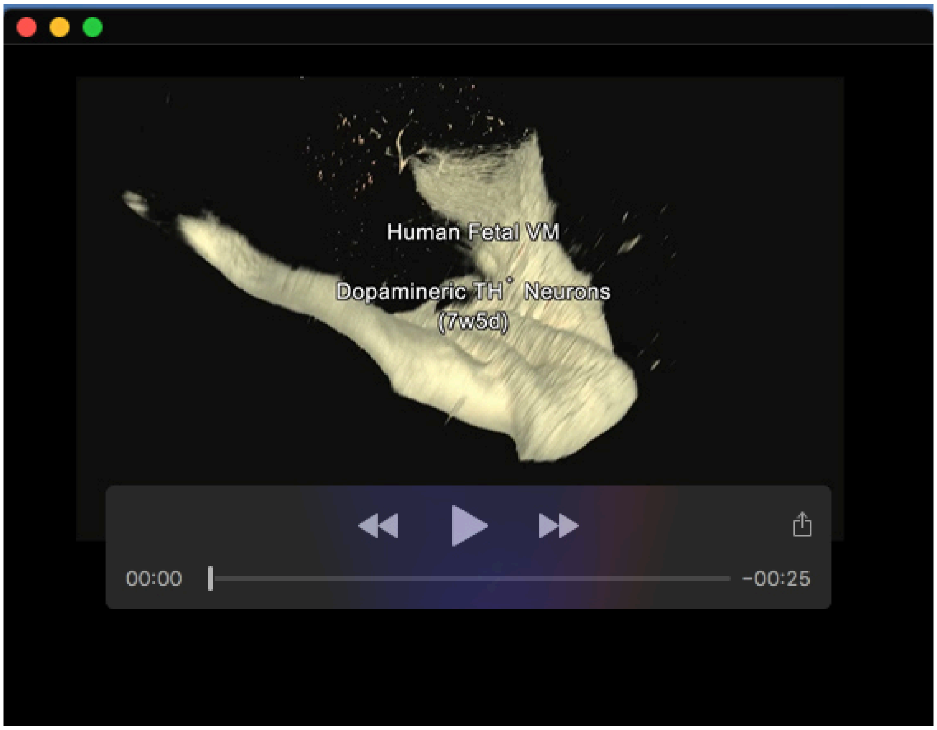

Movie 1. Video reconstruction of iDISCO on 7w5d old human fetal VM.

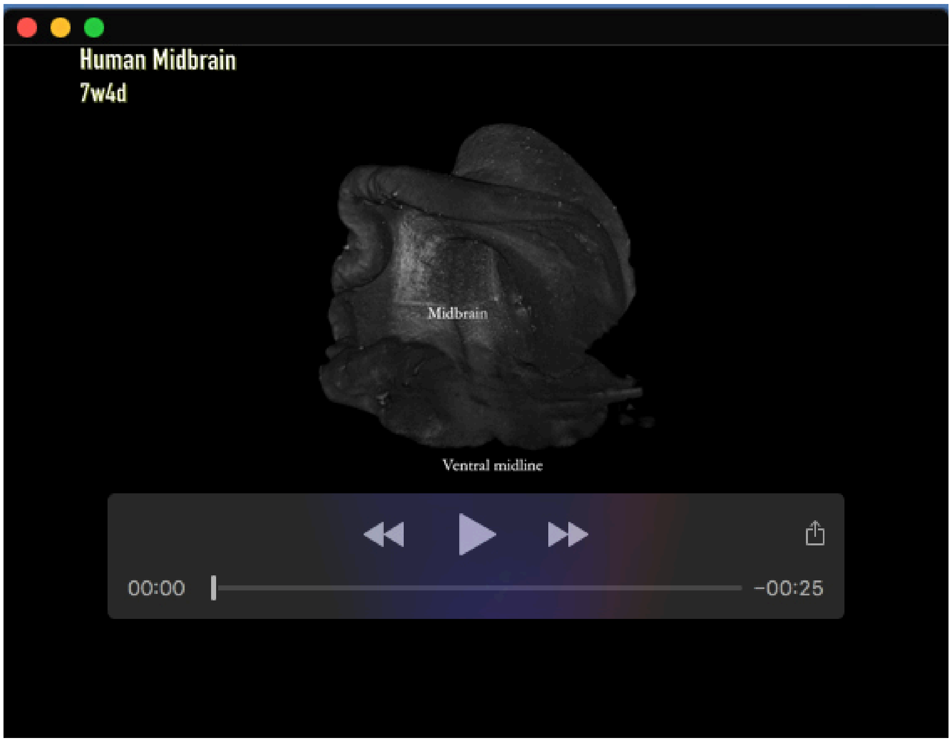

Movie 2. Video reconstruction of iDISCO on 7w4d old huma fetl VM.

Development • Supplementary information

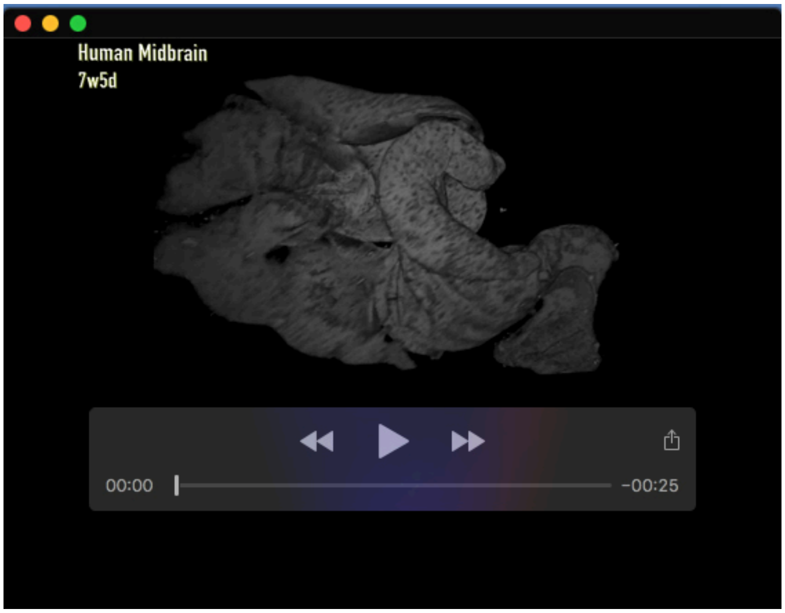

Movie 3. Video reconstruction of iDISCO on 7w5d old human fetal VM.

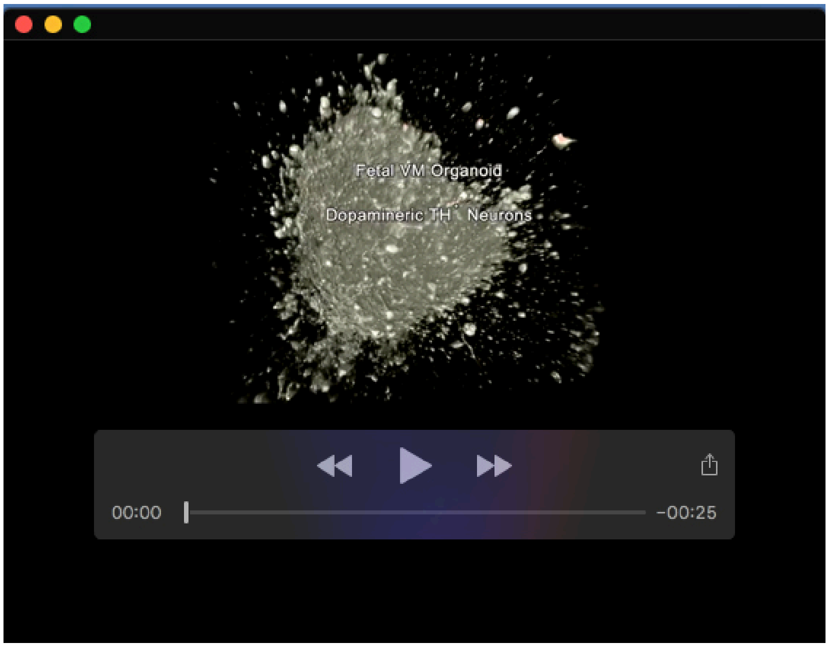

Movie 4. Video reconstruction of iDISCO on 3D human fetal VM cultures.

Development • Supplementary information

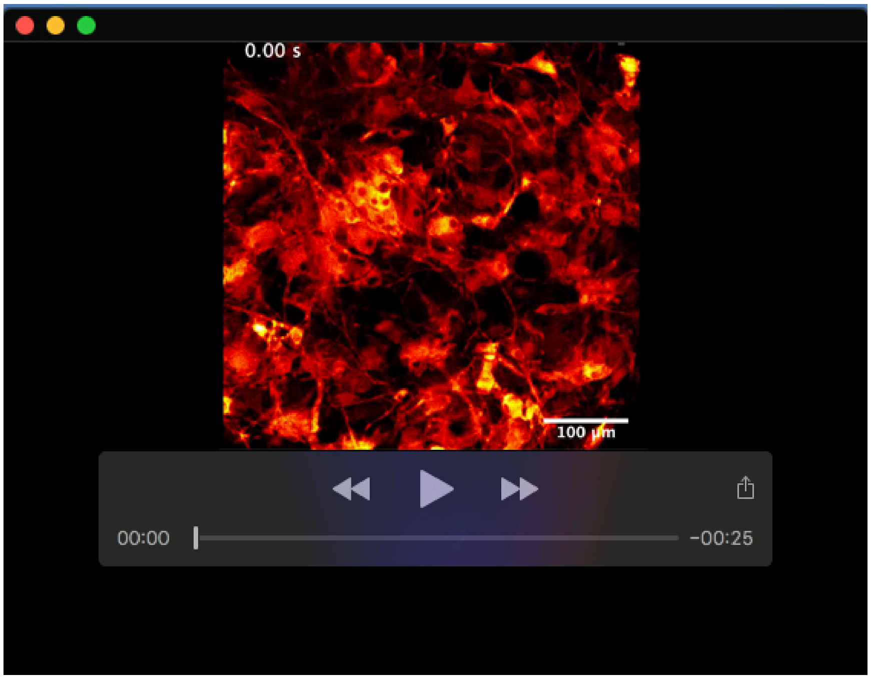

Movie 5. Calcium imaging of 3-month-old 3D human fetal VM cultures.

Development • Supplementary information
