# Supplementary material for: Single-cell transcriptional and functional analysis of dopaminergic neurons in organoid-like cultures derived from human fetal midbrain
Source: Development. 2022 Dec 8;149(23):dev200504. doi: 10.1242/dev.200504 (PMC10114107; doi:10.1242/dev.200504)
Supplement: Supplementary file 1 [file develop-149-200504_review_history.pdf]

Single-cell transcriptional and functional analysis of dopaminergic neurons in organoid-like cultures derived from human fetal midbrain

Marcella Birtele, Petter Storm, Yogita Sharma, Janko Kajtez, Jenny Nelander Wahlestedt, Edoardo Sozzi, Fredrik Nilsson, Simon Stott, Xiaoling L. He, Bengt Mattsson, Daniella Rylander Ottosson, Roger A. Barker, Alessandro Fiorenzano and Malin Parmar
DOI: 10.1242/dev.200504

Editor: Samantha Morris

Review timeline

Original submission:	13 January 2022
Editorial decision:	14 February 2022
First revision received:	19 May 2022
Editorial decision:	20 July 2022
Second revision received:	6 September 2022
Accepted:	12 September 2022

Original submission

First decision letter

MS ID#: DEVELOP/2022/200504

MS TITLE: Single-cell transcriptional and functional analysis of human dopamine neurons in 3D fetal ventral midbrain organoid-like cultures

AUTHORS: Marcella Birtele, Petter Storm, Yogita Sharma, Janko Kajtez, Jenny Nelander Wahlestedt, Edoardo Sozzi, Fredrik Nilsson, Simon Stott, Xiaoling L He, Bengt Mattsson, Daniella Rylander Ottosson, Roger A. Barker, Alessandro Fiorenzano, and Malin Parmar

I have now received all the referees' reports on the above manuscript, and have reached a decision. The referees' comments are appended below, or you can access them online: please go to BenchPress and click on the 'Manuscripts with Decisions' queue in the Author Area.

As you will see, the referees express considerable interest in your work, but have some significant criticisms and recommend a substantial revision of your manuscript before we can consider publication. If you are able to revise the manuscript along the lines suggested, which may involve further experiments, I will be happy receive a revised version of the manuscript. Your revised paper will be re-reviewed by one or more of the original referees, and acceptance of your manuscript will depend on your addressing satisfactorily the reviewers' major concerns. Please also note that Development will normally permit only one round of major revision.

We are aware that you may be experiencing disruption to the normal running of your lab that make experimental revisions challenging. If it would be helpful, we encourage you to contact us to discuss your revision in greater detail. Please send us a point-by-point response indicating where you are able to address concerns raised (either experimentally or by changes to the text) and where you will not be able to do so within the normal timeframe of a revision. We will then provide further guidance. Please also note that we are happy to extend revision timeframes as necessary.

© 2022. Published by The Company of Biologists under the terms of the Creative Commons Attribution License (https://creativecommons.org/licenses/by/4.0/).

Please attend to all of the reviewers' comments and ensure that you clearly highlight all changes made in the revised manuscript. Please avoid using 'Tracked changes' in Word files as these are lost in PDF conversion. I should be grateful if you would also provide a point-by-point response detailing how you have dealt with the points raised by the reviewers in the 'Response to Reviewers' box. If you do not agree with any of their criticisms or suggestions please explain clearly why this is so.

Reviewer 1

Advance summary and potential significance to field

Understanding how different sub-populations of DA neurons arise is critical to the refined development of differentiation protocols for cell therapies in PD. Here, the authors use scRNAseq to analyse 6-11/12 wk fetal VM tissues, and then go on to compare 2D cultures with 3D organoids as potential models of VM maturation. This work adds to our understanding of the molecular profiles and developmental trajectory of authentic dopamine neurons from week 6-11/12, which is necessary for developing better differentiation protocols. The primary finding in this dataset is the usefulness of 3D organoids in maintaining/maturing TH+ neurons in culture long-term. Overall, the manuscript is clearly written and accessible. There is no doubt these data and the use of these techniques will be of interest to the scientific community.

Comments for the author

A primary caveat of the paper is the lack of validation that a 3D organoid actually matures in a manner identical to human VM in situ. In the short term (15-30d) there seems to be good maintenance of TH+ neurons in the 3D organoids. However, they are not mature enough to harbour mature markers capable of distinguishing A9 vs A10 such as Girk2/KCNJ6 (although ALDH1A1 is noted), so significant maturation profiles/trajectories cannot be compared between organoids and human development. Indeed, there also isn't a comparison of human VM at 13-14 weeks to compare to d30 organoids.

Thus, the emphasis here should be on the development of this model organoid system and the potential to use it model human VM. However, the caveats should be noted in the discussion that there would need to be unbiased gene expression analysis of this system long term and direct comparison to human brain to validate organoids as actually depicting genuine development. It may be necessary to use alternative sequencing strategies, such as single nucleus RNAseq.

This should also be clarified in the abstract and Line 213: "Overall, these findings validate our 3D culture system as a functional model able to mimic later stages of fetal DA neuron development in vitro." Since this cannot be concluded here without direct validation with human VM at the equivalent stage (eg 13-15 wks PC), it could instead be said that 3D organoid cultures are superior to 2D cultures in terms of their ability to generate and maintain mature DA neurons in culture, and they have the potential to be used to study VM development.

A second query is why group 6-11/12 week foetuses were all grouped for the initial analysis in Figure 1? A 6 week window that spans early to mid development of this region and encompasses stages that are both useful and not useful for transplantation are included. It seems that relevant data could be obtained and presented for each developmental stage (e.g. every 2 week block) individually to clarify the different signatures of development. Figure 1K could still be generated from the individual datasets to show the developmental profile of the midbrain.

Minor corrections:

Please include the CRLs of all the fetuses – this should be the standard data reported to ensure consistency across labs/studies and to reduce errors based on use of 'post-conception vs gestation'. Additionally, the post-conception time is sometimes stated as 6-11 weeks, and sometimes s 6-12 weeks, therefore, this should be clarified.

Supp Figure 3b – could an image of TH be used for d30? The different histology for the 2 time points makes it difficult to compare them.

© 2022. Published by The Company of Biologists under the terms of the Creative Commons Attribution License (https://creativecommons.org/licenses/by/4.0/).

Figure 3d bottom right panel needs d30 label.

Figure 3/Supp Fig 3. Both Figures show d15 TH/FoxA2, so it is not clear why this image is in both. The axis label is missing on Fig 4B,E.

In the microscopy section, I assume the authors mean 200x and 630x magnification? (Rather than 20 or 63x).

Reviewer 2

Advance summary and potential significance to field

The study by Birtele et al profiles transcriptional signature of individual cells from the first trimester developing human ventral midbrain, revealing profiles for floor plate progenitors, radial glia, early dopaminergic serotonergic/glutamatergic neurons, and oculomotor/trochlear nucleus neurons as well as progenitors and non-neural cells. Because these cells are difficult to access in the second trimester, this study establishes 2D and 3D culture conditions to extend their maturation finding that the 3D approach preserves functional DA neurons.

This study is significant for providing a new resource of single cell gene expression in clinically-relevant cell types that are difficult to access, for providing an approach for studying human DA neurons ex vivo as they mature potentially including primate-specific populations not present in mouse, and for exploring culture methods that support new grafting strategies.

Comments for the author

1) How functionally mature are the DA neurons? A major claim of the study is to provide a transcriptional profile of functionally mature human DA neurons that can be used to guide therapies. However, there is limited maturation and diversification of the DA neurons in this study (no differentiation of CALB and GIRK2 clusters in the 3D culture after 30 days).

The study notes that the 3D cultures can be maintained for longer periods (the 3D cultures were maintained for 95 days for Ca imaging). Would it be possible to stain these more mature cultures for specific subtypes (including GIRK2, CALB previously observed in organoids by this group). Even without single cell profiles, this proof-of-principle could support further emphasis of the significant finding that the 3D culture improves the quality of the DA neurons.

2) The study could benefit from a table showing which samples were used for which analyses (and at which day in culture). For example, the 2D cultures were generated from 8,9, and 10 pc weeks, but need clarity on whether the 3D cultures came from the same individuals and are directly comparable. This clarity would also help the reader interpret the contrast between performance of 2D and 3D cultures.

3) For the cell types themselves, what is the difference between DA-1 and DA-2. Is DA-1 glutamatergic or does the cluster contain a mixture of cell types, including glutamatergic neurons, and a mixture maturation states. Are the DA-1 and DA-2 distinctions related to adult DA neuron subtypes? The diffusion mapping is helpful for interpreting early features of differentiation, but the authors could try to further interpret more mature DA clusters based on adult data (eg from reference 23), which might enable identification of early signatures of adult cell types in the data.

Minor comments:

- Line 55 – describes primate-specific genes for ref 23 – but the reference is to a primate-specific cell type, characterized by a distinct combination of genes – this should be clarified.

-A portion of the data published here was already analyzed (referencing the preprint of the submitted mansucript) in Fiorenzano et al., 2021. However, I noted that only the dopamine clusters were analyzed in the other study and this analysis is more in-depth and designed to present the resource.

© 2022. Published by The Company of Biologists under the terms of the Creative Commons Attribution License (https://creativecommons.org/licenses/by/4.0/).

Consider revising cell type abbreviations (Dopa N and Perot are note widely used).

Does the Serot/Glut cluster contain multiple cell types mixed together?

Figure 1C: This panel shows extensive projections even at early developmental stages. However, the panel needs better labeling and a scale bar. This panel could be oriented to match panel 1A and a box on 1A or a cartoon of the whole brain could better explain the location, or the panel could be removed as the study does not focus on connectivity.

Figure 1E: There is a discrepancy between the UMAP plots and bar chart. See RG-1 and 2.
Also, based on UMAP there are RBCs and Microglia at all timepoints - never 0% as stated in text.

Figure 1G: Needs a color scale to interpret the intensity. Is there a lower level of FOXA2/LMX1A in FP progenitors compared to DA neurons and is this expected or could this indicate that the progenitor cluster is heterogeneous and not exclusively DA neuron producing?

Figure 3 – line 180 cites 3H as showing repetitive firing, but must refer to a different panel. In what ways specifically are these physiological measures typical of a mature DA neuron – the text could provide context and references for interpreting this interpretation.

Figure 4 - The text or legend should also include a description of how cells were selected as dopamine neurons (and progenitors).

Where does the number 12600 come from - is that all cells in the 3D cultures, all DA neurons + progenitors, or specifically all DA neurons?

Is DA-Prog-1 cluster really a DA producing cluster – the cells seem to express minimal FOXA2.

-What is the possible explanation for so few astrocytes in the 3D culture?

-were the cultured cells analyzed together with acute fetal cells to further reconstruct pseudotime?

-Is the GEM and AGTR1 expression restricted to a specific cluster?

Reviewer 3

Advance summary and potential significance to field

Summary of paper:

In this study, Birtele et al demonstrate the sustained production of dopaminergic neurons from human ventral midbrain primary cultures maintained as 3D organoid-like structures. The stated purpose of this study is to develop better in vitro model systems of DA neuron development, specification, and function for use in disease modeling, therapy screening, and ultimately, potential cell-based therapy

Overall this study provides a narrative that details specific limitations with 2D culture systems in accurately recapitulating the development of the human VM, and describes a 3D culture system that appears to more accurately reflect true developmental fate specification of DA neurons. The study makes this claim on the basis of a number of shared marker genes between DA neuron subtypes across culture systems, but falls a bit short. Claims of in vitro similarity to the in vivo standard are hard to demonstrate for sure, but the authors have generated very good quality single cell data that should allow them to perform a significantly deeper dive into comparing the cultured cell types to their in vivo counterparts. In this sense, marker gene analysis is just unfortunately too superficial. This reviewer would like to see a deeper dive into the claims of similarity AND an exploration of how the culture systems actually _differ_ from the in vivo condition which may be just as useful to know prior to adoption of this platform and culture system. Broadly speaking, the models and data that the authors have generated are strong, and no additional experiments are

© 2022. Published by The Company of Biologists under the terms of the Creative Commons Attribution License (https://creativecommons.org/licenses/by/4.0/).

suggested as being required for this study, but the manuscript could benefit from a significantly deeper analysis of the similarities and differences between modalities other than just marker gene analysis.

Comments for the author

Major concerns:

* It is unclear what the addition of the iDISCO cleared LSM images and videos of the hVM are adding to the story of the development of the 3D culture? Their addition as the first part of the results is inconsistent with the narrative established in the introduction, and there is little to no 'interpretation' of these results that provides meaningful context or baseline for interpretation later in the manuscript.

* The authors do a commendable job of demonstrating the the DA neurons that are produced in the 3D organoid cultures share established marker gene expression, setting a baseline comparison for the similarity of the in vitro-derived neurons to their in vitro counterpart. However the authors do not take full advantage of the single cell datasets that they have generated to establish a better validation of their culture system. Marker genes alone are often insufficient to describe the transcriptional state or type of a cell and with single cell analysis, learned patterns of gene co-expression (such as those produced by commonly used dimensionality reduction tools like NMF, ICA, etc) provide a much richer 'signature' of cell types and states and should be explored and compared between the in vitro and in vivo datasets to evaluate their similarity beyond just common marker genes.

* Given that the stated goal of this study is to develop a robust in vitro system for the generation of clinically-relevant DA neurons, it would be exceptionally prudent for the authors to discuss the ways in which the DA neurons produced by their culture system _differ_ from their in vivo counterparts. Again, the authors do spend the bulk of the manuscript describing the similarities, but the differences may also be important. (And discussing these differences should in NO WAY influence the acceptance or publication of this manuscript, but should instead be conducted in the spirit of transparency and the mutual benefit that this information would provide future users of this system). For example, if there are clinically relevant gene expression signatures of PD that are markedly different or differential expression of key genes or processes implicated in PD pathology, these should be reported. At a bare minimum, an exploration of exactly what the differences are (either by DE gene analysis or latent space analysis) is highly pertinent to the relevance of the authors' culture system for future use.

* Better mapping of DA neuron subtypes from 3d culture to A9-A10 subtypes? The authors intimate that the DA-1 and DA-2 subtypes may map to the A9-A10 DA neuron subtypes. If this is meant to be an assertion, then it should be followed up with a bit more comparison and contrast between the experimental modalities to support this interpretation.

* This reviewer is acutely aware of the implications of asking for additional experiments. That being said this is not a requirement for acceptance, but it would be a huge benefit to validate (via smFISH, IF, etc) some of the gene findings from the 3D culture system in vivo including:

** Non-canonical marker genes for DA neuron subtypes that were 'discovered' in the 3D system can be tested for in vivo expression (given availability of tissue which may be exceptionally limiting)

** Non-canonical marker genes for DA neuron subtypes that were 'discovered' in vivo and confirming that they are preserved in the 3D culture system

** Specific genes that were identified as differential between the 3D culture system and in vivo DA neuron subtypes (if any).

* The depth of sequencing appears very light for the number of cells in this study, and the requirement for a minimum of 200 detectable genes suggest that the information content of most cells is relatively low. No recommendations here as it is not feasible to sequence to a greater

© 2022. Published by The Company of Biologists under the terms of the Creative Commons Attribution License (https://creativecommons.org/licenses/by/4.0/).

depth, but this is a general concern for this reviewer, especially if the stated focus of the study is to examine the extent of the heterogeneity of DA neuron subtypes.

* Given that the single cell analyses form the crux of the results of this manuscript, the choice of workflows and tools are relatively narrow. There is a great deal more relevant information that can be gleaned from these datasets that would address the stated goals of the authors in a more comprehensive way. The parameter space(s) for the chosen workflow are not fully explored or even justified and may influence certain interpretations (e.g. number of cellular subtypes/states). For example, the distinction between DA-1 and DA-2 subtypes is not a clear delineation and the UMAP indicates that these two 'subtypes' may actually be a continuous population of cells that have been arbitrarily divided into two subtypes.

* "We employed graph-based clustering to subcluster the DA population, which was found to be further segregated into four distinct molecular identities (Fig. 4H)." Simply because a clustering algorithm split these data into four clusters does not make them 'distinct molecular identities'. In fact, none of these four clusters are 'distinct'. They represent almost arbitrary segmentations of the authors' dataset.

* The authors state on line 232 "…newly found in a human DA neuronal dataset,…" but do not provide a reference for this dataset.

* It is unclear whether the authors are suggesting that the DAprog-1 population identified in the 3D culture system is acting as a similar progenitor population as the RG-1 population in vivo. If this is so, then a transcriptional comparison should be made between the two populations. If this is not the case, then the authors should describe how they think the developmental trajectories differ between the two experimental modalities.

First revision

<u>Author response to reviewers' comments</u>

Reviewer 1 Advance Summary and Potential Significance to Field:
Understanding how different sub-populations of DA neurons arise is critical to the refined development of differentiation protocols for cell therapies in PD.
Here, the authors use scRNAseq to analyse 6-11/12 wk fetal VM tissues, and then go on to compare 2D cultures with 3D organoids as potential models of VM maturation. This work adds to our understanding of the molecular profiles and developmental trajectory of authentic dopamine neurons from week 6-11/12, which is necessary for developing better differentiation protocols. The primary finding in this dataset is the usefulness of 3D organoids in maintaining/maturing TH+ neurons in culture long-term. Overall, the manuscript is clearly written and accessible. There is no doubt these data and the use of these techniques will be of interest to the scientific community.

We thank the Reviewer for their overall positive comments on the work presented. Their careful and insightful remarks and suggestions have enabled us to considerably improve the quality of the manuscript. Please find below a point-by-point response to all comments.

A primary caveat of the paper is the lack of validation that a 3D organoid actually matures in a manner identical to human VM in situ. In the short term (15-30d) there seems to be good maintenance of TH+ neurons in the 3D organoids.
However, they are not mature enough to harbor mature markers capable of distinguishing A9 vs A10 such as Girk2/KCNJ6 (although ALDH1A1 is noted), so significant maturation profiles/trajectories cannot be compared between organoids and human development. Indeed, there also isn't a comparison of human VM at 13-14 weeks to compare to d30 organoids.
Thus, the emphasis here should be on the development of this model organoid system and the potential to use it model human VM. However, the caveats should be noted in the discussion that there would need to be unbiased gene expression analysis of this system long term and

© 2022. Published by The Company of Biologists under the terms of the Creative Commons Attribution License (https://creativecommons.org/licenses/by/4.0/).

direct comparison to human brain to validate organoids as actually depicting genuine development.

We fully agree with the issues raised by the Reviewer and the limitations of the dataset which is due to very restricted access of human fetal tissue. In addition, our ethical permit for sourcing tissue is restricted to week 6-12 and it is not possible for us to source later fetuses. We have now described these limitations better in the manuscript. Additionally, there are some observations from the 3D cultures that we would not expect to match human VM at 13-14 weeks but instead match more mature neurons. For example, we detect functionally active DA neurons expressing DAT, CALB, and GIRK2 already after 30 days in the 3D cultures while the human fetal VM DA neurons has not reached this stage buy the corresponding gestational timepoints. We have now included this point in the Discussion on page 11, 12 of the new version of the manuscript.

It may be necessary to use alternative sequencing strategies, such as single nucleus RNAseq.

We thank the Reviewer for raising this point. Even though snRNA-seq are often superior for capturing neurons when isolating cells from the adult brain, we do capture more than 14 000 DA neurons including very mature neurons in our scRNAseq data set. Hence the main problem relating to lack of neurons recovered from the adult brain when performing scRNAseq does not seem to be a major issue when isolating the cells from organoids or fetal brain. We have made a note on this in the revised version of the manuscript on page 10.

In addition, it should not be forgotten that snRNA-seq comes with problems on its own, including reduced gene recovery and potential bias in cell types recovered (https://genomebiology.biomedcentral.com/articles/10.1186/s13059-020-02048-6) which we now also comment on in the revised version of manuscript on page 10.

This should also be clarified in the abstract and Line 213: "Overall, these findings validate our 3D culture system as a functional model able to mimic later stages of fetal DA neuron development in vitro." Since this cannot be concluded here without direct validation with human VM at the equivalent stage (eg 13-15 wks PC), it could instead be said that 3D organoid cultures are superior to 2D cultures in terms of their ability to generate and maintain mature DA neurons in culture, and they have the potential to be used to study VM development.

We agree with the Reviewer and have now modified the Abstract based on their suggestion.

A second query is why group 6-11/12 week foetuses were all grouped for the initial analysis in Figure 1? A 6 week window that spans early to mid development of this region and encompasses stages that are both useful and not useful for transplantation are included. It seems that relevant data could be obtained and presented for each developmental stage (e.g. every 2 week block) individually to clarify the different signatures of development. Figure 1K could still be generated from the individual datasets to show the developmental profile of the midbrain.

We apologize for any lack of clarity that caused confusion with regard to the grouped samples analyzed.

In this study, the samples were initially grouped to enable analyze of as high number of cells as possible in order to provide a more accurate identification/assignment of the different cell types, and also to more accurately identify differences and similarities. This has now been clarified in the legend of figure 1 (page 18) and in the materials and methods (page 15).

After this grouping and cell type assignment, we report the samples divided by week, showing individual UMAP plots (Figure 1E) and quantifying cell types per time point (Figure 1E). We have now clarified this in the text in methods and figure legend. In the revised version, we have added pseudotime diffusion plots for the different developmental stages in Supplementary Figure 1F to show the pseudotemporal development for each sample more clearly, and that mature cell types come from later time points.

© 2022. Published by The Company of Biologists under the terms of the Creative Commons Attribution License (https://creativecommons.org/licenses/by/4.0/).

Minor corrections:
Please include the CRLs of all the fetuses - this should be the standard data reported to ensure consistency across labs/studies and to reduce errors based on use of 'post- conception vs gestation'. Additionally, the post-conception time is sometimes stated as 6- 11 weeks, and sometimes s 6-12 weeks, therefore, this should be clarified.

We are grateful to the Reviewer for identifying the inconsistencies in the text. We have ethical permit to collect embryos in the range of 6-12 weeks but in this study have only used 6-11 weeks. We have now corrected the text to state that the samples used in the study are 6–11 weeks post conception in all sections.

In addition we now include a supplementary table (Supplementary Table 1) providing details of the CRLs and developmental stage of all the fetuses used in the study.

Supp Figure 3b - could an image of TH be used for d30? The different histology for the 2 time points makes it difficult to compare them.

We have now included AADC/TH immunohistochemistry at day 15 and day 30 for a direct comparison in new Supplementary Figure 3A.

Figure 3d bottom right panel needs d30 label.

We have corrected the figure labeling.

Figure 3/Supp Fig 3. Both Figures show d15 TH/FoxA2, so it is not clear why this image is in both.

We thank the Reviewer for highlighting the unclear presentation of this point. We have now reorganized the figures so that the TH/FOXA2 image is displayed only once in the new main Figure 3.

The axis label is missing on Fig 4B,E.

We have inserted the axis label for Figures 4B and E.

In the microscopy section, I assume the authors mean 200x and 630x magnification? (Rather than 20 or 63x).

20x and 63x are the objectives used for image acquisition. We have now clarified this in the Methods section.

Reviewer 2 Advance Summary and Potential Significance to Field:
The study by Birtele et al profiles transcriptional signature of individual cells from the first trimester developing human ventral midbrain, revealing profiles for floor plate progenitors, radial glia, early dopaminergic, serotonergic/glutamatergic neurons, and oculomotor/trochlear nucleus neurons as well as progenitors and non-neural cells. Because these cells are difficult to access in the second trimester, this study establishes 2D and 3D culture conditions to extend their maturation finding that the 3D approach preserves functional DA neurons.
This study is significant for providing a new resource of single cell gene expression in clinically-relevant cell types that are difficult to access, for providing an approach for studying human DA neurons ex vivo as they mature, potentially including primate- specific populations not present in mouse, and for exploring culture methods that support new grafting strategies.

We are grateful to the Reviewer for their generally positive comments. The quality of the manuscript has been greatly improved thanks to their insightful remarks and suggestions. Below is a point-by-point response detailing the changes made.

Reviewer 2 Comments for the Author:
1) How functionally mature are the DA neurons? A major claim of the study is to provide a transcriptional profile of functionally mature human DA neurons that can be used to guide

© 2022. Published by The Company of Biologists under the terms of the Creative Commons Attribution License (https://creativecommons.org/licenses/by/4.0/).

therapies. However, there is limited maturation and diversification of the DA neurons in this study (no differentiation of CALB and GIRK2 clusters in the 3D culture after 30 days).

The study notes that the 3D cultures can be maintained for longer periods (the 3D cultures were maintained for 95 days for Ca imaging). Would it be possible to stain these more mature cultures for specific subtypes (including GIRK2, CALB previously observed in organoids by this group). Even without single cell profiles, this proof-of-principle could support further emphasis of the significant finding that the 3D culture improves the quality of the DA neurons.

In the original version, we show the expression of TH/CALB and TH/DAT to show the phenotypic maturity of the DA neurons after 30 days in the 3D cultures at which point they are electrophysiologically active. Based on the reviewers' comment, we have now also included a staining of TH/GIRK2 at this timepoint in new Fig 3D.

To support the claim that DA neurons can be maintained for longer periods, we have now included DAT/ALDH1a1 immunohistochemistry after 100 days in culture in new Fig 3E, right panel.

2) The study could benefit from a table showing which samples were used for which analyses (and at which day in culture). For example, the 2D cultures were generated from 8,9, and 10 pc weeks, but need clarity on whether the 3D cultures came from the same individuals and are directly comparable. This clarity would also help the reader interpret the contrast between performance of 2D and 3D cultures.

We have now inserted a table (Supplementary Table 1) in the supplementary material where we report details of the embryos, including the development stage from which we started generating the 2D and 3D cultures. Additionally, we have now clarified in the Methods section that VM tissue dissected from the same human embryo was split in two to be used for both fetal culture systems described.

3) For the cell types themselves, what is the difference between DA-1 and DA-2. Is DA-1 glutamatergic or does the cluster contain a mixture of cell types, including glutamatergic neurons, and a mixture maturation states. Are the DA-1 and DA-2 distinctions related to adult DA neuron subtypes? The diffusion mapping is helpful for interpreting early features of differentiation, but the authors could try to further interpret more mature DA clusters based on adult data (eg from reference 23), which might enable identification of early signatures of adult cell types in the data.

We thank the Reviewer for raising this point that was discussed in the original manuscript (lines 217-253) we have now expanded this section further. We have now added a silhouette analysis to further interpret and validate the clustering of dopamine subtypes. Indeed, four clusters were shown to give the highest average silhouette score, indicative of a cells being well matched to its own cluster and poorly matched to neighboring clusters (Supplementary Figure 4B). Average silhouette scores per cluster indicated that DA-prog1 and DA-2 were the most robust whereas plasticity might exist between DA-prog2 and DA-1.

This analyses also shows the absence of/lack of cell type mixtures in the DA-1 cluster. To address this point in more detail, we have now also added a dot-plot showing gabaergic and glutamatergic markers among the identified DA subclusters (Supplementtray Fig 4F). As shown, the minimal expression of these markers in all 4 identified clusters suggests that the dopaminergic compartment is predominant in 3D cultures. More likely DA-1 concomitantly expresses dopamine neuron machinery as well as some genes related to glutamatergic neurons, as also shown in the mouse scRNAseq dataset from *Tiklova et al* (Nature Communication 2020).

Minor comments:

- Line 55 - describes primate-specific genes for ref 23 - but the reference is to a primate-specific cell type, characterized by a distinct combination of genes - this should be clarified.

We apologize for the error. We have now corrected the wording referring to the citation.

© 2022. Published by The Company of Biologists under the terms of the Creative Commons Attribution License (https://creativecommons.org/licenses/by/4.0/).

-A portion of the data published here was already analyzed (referencing the preprint of the submitted mansucript) in Fiorenzano et al., 2021. However, I noted that only the dopamine clusters were analyzed in the other study and this analysis is more in-depth and designed to present the resource. Consider revising cell type abbreviations

The previous study focused entirely on midbrain organoid differentiation from human pluripotent stem cells and did not report any of the data included in this current manuscript, However, the fetal VM dataset was used a very valuable tool to confirm the presence of authentic DA molecular identities in the organoids established from pluripotent stem cells in the Fiorenzano et al, 2021 study.

We have now used Sert and DA as abbreviations for serotonergic and dopaminergic neurons, respectively, to be consistent with the names of previously identified clusters.

Does the Serot/Glut cluster contain multiple cell types mixed together?

We apologize for the typo, which has now been corrected. The cluster only refers to the serotonergic cluster.

Figure 1C: This panel shows extensive projections even at early developmental stages. However, the panel needs better labeling and a scale bar. This panel could be oriented to match panel 1A and a box on 1A or a cartoon of the whole brain could better explain the location, or the panel could be removed as the study does not focus on connectivity.

We agree with the comment and have now better reported anterior/posterior orientation of the panel 1C and we added VM midline with a red dash and included the scale bar. We have also reported in the figure legend the orientation in Figure 1A (sagittal view), Figure 1B (anterior view) and Figure 1C (dorsal view).

Figure 1E: There is a discrepancy between the UMAP plots and bar chart. See RG-1 and 2.
Also, based on UMAP there are RBCs and Microglia at all timepoints - never 0% as stated in text.

We thank the reviewer for spotting this and apologize for this mistake. We have now included the correct bar chart and reported the correct percentage (as minimum and maximum value found across samples) in the text.

Figure 1G: Needs a color scale to interpret the intensity. Is there a lower level of FOXA2/LMX1A in FP progenitors compared to DA neurons and is this expected or could this indicate that the progenitor cluster is heterogeneous and not exclusively DA neuron producing?

We are grateful to the Reviewer for pointing out this issue. We have now added the scale intensity bar.

It´s known that post-mitotic DA neurons still retain the expression of *FOXA2* and *LMX1A*. We were not surprised to see a greater expression of both genes in the DA cluster rather than in the FPP. We believe that the FPP cluster that we captured in the single cell analysis is mostly composed of early floor plate progenitor cells, given the high enrichment of *WNT5A* and *SHH* expression.

Figure 3 - line 180 cites 3H as showing repetitive firing, but must refer to a different panel. In what ways specifically are these physiological measures typical of a mature DA neuron - the text could provide context and references for interpreting this interpretation.

We have now corrected the figure reference in the text and also included references to previously published works describing the electrophysiological properties of DA neurons in order to provide a better context for the reported results and their interpretation.

Figure 4 - The text or legend should also include a description of how cells were selected as dopamine neurons (and progenitors).

© 2022. Published by The Company of Biologists under the terms of the Creative Commons Attribution License (https://creativecommons.org/licenses/by/4.0/).

We have used the dataset from *La Manno et al* (Cell, 2017) combined with label transfer from the uncultured data set (i.e. figure 1) as a reference for the cell-type assignment in our VM cultures. We have now better clarified this information in the figure legend and materials and. methods.

Where does the number 12600 come from - is that all cells in the 3D cultures, all DA neurons + progenitors, or specifically all DA neurons?

The number of 12600 cells refers only to the DA neurons analyzed from 3D culture. We have now better clarified this information throughout the text.

Is DA-Prog-1 cluster really a DA producing cluster – the cells seem to express minimal FOXA2.

This is an interesting observation. While the *FOXA2* is reduced the cluster express other key markers such as *SOX2*, *SOX6 and NR4A2* we believe that this cluster exhibits the molecular features of DA progenitors in the transition from floor plate cells to DA neurons. We have elaborated in this in the text on page 8 (line 244-247).

-What is the possible explanation for so few astrocytes in the 3D culture?

We thank the Reviewer for raising this interesting point. We do not have any definitive answers, but believe that 3D culture generates more "VM-like" conditions, preserving the expansion and maturation of fetal DA neurons over time. Conversely, the 2D culture conditions seem to favor glia proliferation and thus take over the culture at the expense of neurons.

-were the cultured cells analyzed together with acute fetal cells to further reconstruct pseudotime?

We have now provided pseudotime analysis including both culture and uncultured VM (Supplementary Fig1G).

-Is the GEM and AGTR1 expression restricted to a specific cluster?

We found *GEM* and *AGTR1* highly enriched in DA neurons, but their expression was not restricted to this cluster.

Reviewer 3 Advance Summary and Potential Significance to Field:
Summary of paper:
In this study, Birtele et al demonstrate the sustained production of dopaminergic neurons from human ventral midbrain primary cultures maintained as 3D organoid- like structures. The stated purpose of this study is to develop better in vitro model systems of DA neuron development, specification, and function for use in disease modeling, therapy screening, and ultimately, potential cell-based therapy

Overall this study provides a narrative that details specific limitations with 2D culture systems in accurately recapitulating the development of the human VM, and describes a 3D culture system that appears to more accurately reflect true developmental fate specification of DA neurons. The study makes this claim on the basis of a number of shared marker genes between DA neuron subtypes across culture systems, but falls a bit short. Claims of in vitro similarity to the in vivo standard are hard to demonstrate for sure, but the authors have generated very good quality single cell data that should allow them to perform a significantly deeper dive into comparing the cultured cell types to their in vivo counterparts. In this sense, marker gene analysis is just unfortunately too superficial. This reviewer would like to see a deeper dive into the claims of similarity AND an exploration of how the culture systems actually _differ_ from the in vivo condition which may be just as useful to know prior to adoption of this platform and culture system. Broadly speaking, the models and data that the authors have generated are strong, and no additional experiments are suggested as being required for this study, but the manuscript could benefit from a significantly deeper analysis of the similarities and differences between modalities other than just marker gene analysis.

We thank the reviewer for their positive remarks and recognition of the strength and value of our

© 2022. Published by The Company of Biologists under the terms of the Creative Commons Attribution License (https://creativecommons.org/licenses/by/4.0/).

single cell dataset. We are also very grateful to the reviewer for appreciating the difficulty in obtaining human fetal samples and for acknowledging that study presented in the manuscript does not require any further experiments. We have followed the suggestions to improve the quality of manuscript. Please find a point-by-point response to all comments below.

Reviewer 3 Comments for the Author:
Major concerns:
It is unclear what the addition of the iDISCO cleared LSM images and videos of the hVM are adding to the story of the development of the 3D culture? Their addition as the first part of the results is inconsistent with the narrative established in the introduction, and there is little to no 'interpretation' of these results that provides meaningful context or baseline for interpretation later in the manuscript.

We apologize for any lack of clarity. We have now better reported anterior/posterior orientation of the panel 1C and we added VM midline with a red dash and included the scale bar. We have also reported in the figure legend the orientation in Figure 1A (sagittal view), Figure 1B (anterior view) and Figure 1C (dorsal view). We have now also added a comprehensive description of Figure 1C in the Result section (page3, line 79-83).

The authors do a commendable job of demonstrating the DA neurons that are produced in the 3D organoid cultures share established marker gene expression, setting a baseline comparison for the similarity of the in vitro-derived neurons to their in vitro counterpart. However the authors do not take full advantage of the single cell datasets that they have generated to establish a better validation of their culture system. Marker genes alone are often insufficient to describe the transcriptional state or type of a cell and with single cell analysis, learned patterns of gene co-expression (such as those produced by commonly used dimensionality reduction tools like NMF, ICA, etc) provide a much richer 'signature' of cell types and states and should be explored and compared between the in vitro and in vivo datasets to evaluate their similarity beyond just common marker genes.

We agree with the reviewer and have now compared the datasets obtained in vitro and in vivo using single-cell weighted correlation network analysis (scWGCNA), focusing on the occurrence of transcriptome-wide gene co-expression modules in the dopamine neurons. WGCNA, originally developed for the detection of gene co-expression modules in bulk RNA- seq data, has seen a recent surge in popularity, given the high number of replicate (i.e. single cells) available when working with scRNA-seq data. Briefly, we performed (a) pseudocell construction, to increase overall robustness and identification of highly variable genes (b) WGCNA module detection followed by gene ontology (GO)-term enrichment analyses. Topology analysis suggested six gene co-expression modules in the cultured dopamine neurons, labelled as "yellow", "green", "red", "brown", turquoise" and "blue". These modules were then linked to function using enrichment analysis, identifying for example ATF6- mediated UPR response. Finally we compared the module expression in uncultured dopamine neurons, identifying high expression of module "green", "brown", "turquoise" and "blue" in the most mature DA neurons. The manuscript has been updated with this new analysis (page 9, line 274-288).

Given that the stated goal of this study is to develop a robust in vitro system for the generation of clinically-relevant DA neurons, it would be exceptionally prudent for the authors to discuss the ways in which the DA neurons produced by their culture system _differ_ from their in vivo counterparts. Again, the authors do spend the bulk of the manuscript describing the similarities, but the differences may also be important. (And discussing these differences should in NO WAY influence the acceptance or publication of this manuscript, but should instead be conducted in the spirit of transparency and the mutual benefit that this information would provide future users of this system). For example, if there are clinically relevant gene expression signatures of PD that are markedly different or differential expression of key genes or processes implicated in PD pathology, these should be reported. At a bare minimum, an exploration of exactly what the differences are (either by DE gene analysis or latent space analysis) is highly pertinent to the relevance of the authors' culture system for future use.

We thank the reviewer for raising this important point. At the same time, it should be appreciated

© 2022. Published by The Company of Biologists under the terms of the Creative Commons Attribution License (https://creativecommons.org/licenses/by/4.0/).

that doing a genome-wide comparison is not trivial, due to technical batch effects related to sample handling, cell preparation methodologies and sequencing related differences meaning that an identified difference could be due to technical reasons. Nonetheless, we have now made a direct comparison between our dataset and that of post-mortem human brain from *Agarwal et al* Nat Comm 2020. We discuss the points of similarity and differences between cell types in these two datasets in the Result section (page 9, line 294-299) and also provide a list of differentially expressed genes between human post-mortem DAN and our DAN.

Better mapping of DA neuron subtypes from 3d culture to A9-A10 subtypes? The authors intimate that the DA-1 and DA-2 subtypes may map to the A9-A10 DA neuron subtypes. If this is meant to be an assertion, then it should be followed up with a bit more comparison and contrast between the experimental modalities to support this interpretation.

We thank the reviewer for helping us to clarify this point better. Our single cell data suggest that DA-1 and DA-2 may reflect the diversity of A9 and A10 neurons. However, this remains a matter of speculation. We did attempt to make a comparison with single cell datasets currently available, but without success. First of all, single cell datasets concerning human DA diversity are only reported in pre-press papers. It would be a valuable analysis to project our scRNAseq dataset onto adult data reported by Kamath et al's biorXiv. There is also another pre-press paper by Wang et al., recently published in biorXiv which reports a scRNAseq dataset from human DA neurons in the substantia nigra. Unfortunately, the authors did not release the raw data needed to perform a direct comparative analysis between our fetuses and their adult DA neuron subclusters.

* **This reviewer is acutely aware of the implications of asking for additional experiments. That being said, this is not a requirement for acceptance, but it would be a huge benefit to validate (via smFISH, IF, etc) some of the gene findings from the 3D culture system in vivo including:**
**** Non-canonical marker genes for DA neuron subtypes that were 'discovered' in the 3D system can be tested for in vivo expression (given availability of tissue which may be exceptionally limiting)**
**** Non-canonical marker genes for DA neuron subtypes that were 'discovered' in vivo and confirming that they are preserved in the 3D culture system**
**** Specific genes that were identified as differential between the 3D culture system and in vivo DA neuron subtypes (if any).**

We completely agree with the reviewer that it would be very interesting to validate new genes identified *in vivo*. Unfortunately, human post-mortem brain tissue samples are extremely difficult to obtain. In addition to being outside the scope of our study, we also believe that the validation of these genes in the mouse brain would be misleading due to the obvious differences between species (La Manno et al. *Cell,* 2017, Poulin et al., *Trends Neurosci.*, 2021). The study presented in this manuscript specifically aims to generate a transcriptional profile of the developing human fetal VM and functionally mature human DA neurons, proposing 3D fetal culture as a model to authentically recapitulate the later stages of DA differentiation.

The depth of sequencing appears very light for the number of cells in this study, and the requirement for a minimum of 200 detectable genes suggest that the information content of most cells is relatively low. No recommendations here as it is not feasible to sequence to a greater depth, but this is a general concern for this reviewer, especially if the stated focus of the study is to examine the extent of the heterogeneity of DA neuron subtypes.

We appreciate the reviewer for noting this. Even though the minimum for a cell to be included in downstream analysis was >200 genes expressed the average number of genes expressed for the DAN is far higher (1151 genes/cells) as well as for the uncultured data set (1102 genes/cell). We have clarified this in the methods section (page 14). We do, however, agree that a deeper sequencing would have been useful for resolving cells that are close in transcriptome space (i.e. DA subtypes) and have added a comment about this in the manuscript on page 10.

*** Given that the single cell analyses form the crux of the results of this manuscript, the choice of workflows and tools are relatively narrow. There is a great deal more relevant information that can be gleaned from these datasets that would address the stated goals of the authors in a more comprehensive way. <u>The parameter space(s) for the chosen workflow are not fully</u>**

© 2022. Published by The Company of Biologists under the terms of the Creative Commons Attribution License (https://creativecommons.org/licenses/by/4.0/).

explored or even justified and may influence certain interpretations (e.g. number of cellular subtypes/states).

We thank the reviewer for this suggestion. We did an optimization of parameters based on previous experiences but did not include the details. We have now extended the methods section to better describe this. It should be noted though, that varying the most commonly tuned parameters (e.g. normalization method, # of variable genes, dimension of the latent space) didn't materially influence the outcome, which is in line with what has been shown before (https://genomebiology.biomedcentral.com/articles/10.1186/s13059-020-02128-7).

For example, the distinction between DA-1 and DA-2 subtypes is not a clear delineation and the UMAP indicates that these two 'subtypes' may actually be a continuous population of cells that have been arbitrarily divided into two subtypes.

We thank the reviewer for raising this point, which was in fact discussed in the original manuscript (lines 217-253). We have now expanded this section further. We have performed a silhouette analysis to further interpret and validate the clustering of DA subtypes: four clusters were shown to give the highest average silhouette score, indicative of a cells being well matched to their own cluster and poorly matched to neighboring clusters (Supplementary Figure 4B). Average silhouette scores per cluster indicated that DA-prog1 and DA-2 were the most robust, whereas plasticity might exist between DAProg2 and DA1. This analysis also shows the absence/lack of cell type mixtures in the DA-1 cluster. To address this point in more detail, we have now also analyzed the expression of gabaergic and glutamatergic markers in the identified DA subclusters.

"We employed graph-based clustering to subcluster the DA population, which was found to be further segregated into four distinct molecular identities (Fig. 4H)." Simply because a clustering algorithm split these data into four clusters does not make them 'distinct molecular identities'. In fact, none of these four clusters are 'distinct'. They represent almost arbitrary segmentations of the authors' dataset.

We apologize for any lack of clarity. Please see the point above. The wording has also been updated in new version of the manuscript.

The authors state on line 232 "…newly found in a human DA neuronal dataset,…" but do not provide a reference for this dataset.

We thank the reviewer for noticing this omission. We have now inserted the reference for the dataset page 8 line 251-252.

It is unclear whether the authors are suggesting that the DAprog-1 population identified in the 3D culture system is acting as a similar progenitor population as the RG-1 population in vivo. If this is so, then a transcriptional comparison should be made between the two populations. If this is not the case, then the authors should describe how they think the developmental trajectories differ between the two experimental modalities.

This is a really good suggestion. We have now provided a direct comparison between RG-1 and DAprog1 in the new Suppl Fig 4E. These two cell types have many midbrain FP markers in common. However, unlike DA-prog, RG-1 shows a strong proliferative signature (CCNB2, TOP2A, MIKI67) accompanied by expression of canonical RG markers, including MSX1, VIM, and SLC1A3. We have updated the manuscript with this new data (page 8, line 259-264).

© 2022. Published by The Company of Biologists under the terms of the Creative Commons Attribution License (https://creativecommons.org/licenses/by/4.0/).

Second decision letter

MS ID#: DEVELOP/2022/200504

MS TITLE: Single-cell transcriptional and functional analysis of human dopamine neurons in 3D fetal ventral midbrain organoid-like cultures

AUTHORS: Marcella Birtele, Petter Storm, Yogita Sharma, Janko Kajtez, Jenny Nelander Wahlestedt, Edoardo Sozzi, Fredrik Nilsson, Simon Stott, Xiaoling L He, Bengt Mattsson, Daniella Rylander Ottosson, Roger A. Barker, Alessandro Fiorenzano, and Malin Parmar

I have now received all the referees reports on the above manuscript, and have reached a decision. The referees' comments are appended below, or you can access them online: please go to BenchPress and click on the 'Manuscripts with Decisions' queue in the Author Area.

The overall evaluation is positive and we would like to publish a revised manuscript in Development, provided that the referees' comments can be satisfactorily addressed. Please attend to all of the reviewers' comments in your revised manuscript and detail them in your point-by-point response. If you do not agree with any of their criticisms or suggestions explain clearly why this is so. If it would be helpful, you are welcome to contact us to discuss your revision in greater detail. Please send us a point-by-point response indicating your plans for addressing the referee's comments, and we will look over this and provide further guidance.

Reviewer 1

Advance summary and potential significance to field

This paper provides useful and interesting methods to culture organoids of human fetal dopamine neurons. These methods, will no doubt, be of interest to the field.

Comments for the author

The authors have taken on board the vast majority of comments from the reviewers and I commend them for their diligence and the production of a more refined manuscript.
I am pleased with the revised version, with one exception.
The title suggests that these are organoids of fetal "ventral midbrain". However the fact that this cannot be verified by direct comparison to authentic fetal ventral midbrain, and the fact that many differences are likely evident between the organoids and authentic midbrain (ie loss of Sert+ neurons, loss of astrocytes in the organoids), it is not accurate to call these "organoids of ventral midbrain".
They are, instead, something more akin to organoids of human fetal dopamine neurons.

Reviewer 2

Advance summary and potential significance to field

The study by Birtele et al profiles transcriptional signature of individual cells from the first trimester developing human ventral midbrain, revealing profiles for floor plate progenitors, radial glia, early dopaminergic serotonergic/glutamatergic neurons, and oculomotor/trochlear nucleus neurons as well as progenitors and non-neural cells. Because these cells are difficult to access in the second trimester, this study establishes 2D and 3D culture conditions to extend their maturation finding that the 3D approach preserves functional DA neurons.

This study is significant for providing a new resource of single cell gene expression in clinically-relevant cell types that are difficult to access, for providing an approach for studying human DA neurons ex vivo as they mature potentially including primate-specific populations not present in mouse, and for exploring culture methods that support new grafting strategies.

Comments for the author

© 2022. Published by The Company of Biologists under the terms of the Creative Commons Attribution License (https://creativecommons.org/licenses/by/4.0/).

The authors have mainly responded to the suggestions raise. I have largely minor comments
Major comments:
• Line 165 – “at an accelerated pace as would be expected during normal development in the intact fetal VM.” I am unsure what the authors mean by “accelerated pace” – is there empirical data that the rate of change in maturation properties exceeds that expected from earlier stages (or in vivo)? If not, then the word accelerated is not appropriate. And if the pace would expected from in vivo development, then that would also not be accelerated.

Minor comments:
• Line 24 – change to “This approach revealed”
• Line 60 “have” should be “has”
• Lane 80 - explain abbreviation LSM
• Line 101 change to “canonical markers”
• Line 135 – TH-low appears to express canonical intermediate progenitor markers like HES6, DLL1, GADD45G, INSM1, NHLH1, NEUROG2
• Line 143-149 and figure 1K and 1L - there's some mislabeling and the colors have been swapped. The neuroblast cluster is called Prog and the Serotonergic cluster is labeled Glut/Sert N rather than Sert> Maybe wrong version of figure? Fig 1D-E and Supplementary 1F-G have the same labeling and colors.
• Line 151-153 - I personally still think the TH high and low division might be attributed to maturity but I think we can leave this alone
• Figure 3D - new image of GIRK2 but not very clear. Maybe show a zoomed in inset without DAPI?
• Line 192 and figure 3E - new panel in figure 3 could have it's own letter (3F)
• Line 194 – 198 – thank you for adding these helpful references!
• Line 234 – more clarification of the exact criteria for substetting the 12,600 cultured DA neurons would be important and also a defense of using the word “mature” here as less than half of them express TH. Was this actually subsetting the DA lineage vs mature DA neurons?
• Line 247-251 - this new sentence is a bit unlcear to me and I'm not sure I understand correctly - I don't really see NR4A2 expression in DAProg-1 based on figure 4I-J
• Line 299 should clarify, as in the rebuttal that CALB1 and GEM do not appear to be localized to a particular subset of DA neurons
• Line 373 – not sure that Kanton 2019 shows meaningful overlap of maturation signatures with adult brain snRNA-seq vs limited preservation of regional and differentially expressed markers

Reviewer 3

Advance summary and potential significance to field

- Paper is improved from previous version in many, but not all places.
- Specific suggestions are made below that might help to resolve authors' stated inability to respond to specific requests from reviewers.

Comments for the author

• Response generally _acknowledges_ concerns of the reviewers but in a few cases does not _address_ the concerns directly.
• Additional validation and comparison(s) to human VM DA neurons still not as deep as I would like (suggesting that in lieu of smFISH which is a _very_ difficult ask, that authors compare their data a bit more deeply to Wang et al 2022 preprint data; link below)
• Authors have generally taken recommendations for increased clarity and for toning down conclusions to heart.

Specific Response Points:
• The addition of the WGCNA module analysis, as requested, is commendable however, little ‘biological’ interpretation has been made from/with this analysis. Other than identifying GO terms with enrichment in these modules (relatively few of which appear to be relevant or contextually related to DA neurons or their roles/development) the authors provide only a cursory description of how the learned components are ‘used’ in the in vivo dataset. No quantification is described, and

© 2022. Published by The Company of Biologists under the terms of the Creative Commons Attribution License (https://creativecommons.org/licenses/by/4.0/).

no discussion about the biological interpretations of what individual modules represent about the comparative similarities/differences between DA-subtypes in vitro and in vivo. The result here is that the specific analysis was technically ‘performed’ but no meaningful value was extracted from the results (which was the original intent from the reviewer’s ask).

•The specific request for a differential comparison to the in vivo DA neurons (Argwal 2020) is a solid addition, demonstrating primarily that the largest differences in gene expression might simply be attributed to differences in maturation rate.

•The authors indicate that a direct comparison of DA-1/2 subtypes with A9-A10 subtypes is not possible due to low data quality/availability. Specifically, the authors reply that “[a] pre-press paper by Wang et al., recently published in biorXiv which reports a scRNAseq dataset from human DA neurons in the substantia nigra. Unfortunately, the authors did not release the raw data needed to perform a direct comparative analysis between our fetuses and their adult DA neuron subclusters.” While not directly mentioned in the specified preprint, a quick online search indicated that these data are in fact available on the NIH SRA (see link and project accession below). Given the quality, breadth, and depth of these comparable in vivo data, this should make response to the specific request (better mapping of DA-1 and Da-2 subtypes to in vivo subtypes) possible. I leave the requirement for this comparison at the discretion of the authors and editors.
- Accession: PRJNA767088
- ID: 767088
- Deposit Date: 28-Sep-2021
- https://www.ncbi.nlm.nih.gov/bioproject/767088

• Given the authors’ (completely understandable) response that availability of human fetal tissue for smFISH or other validation is severely limited, and the authors’ assertions that their focus “ in this manuscript specifically aims to generate a transcriptional profile of the developing human fetal VM and functionally mature human DA neurons, proposing 3D fetal culture as a model to authentically recapitulate the later stages of DA differentiation…”, I would suggest that a further comparison to the high-depth/quality Wang et all 2022 preprint data would be sufficient additional (and external) validation.
Especially if performed at the DA-neuron subtype level. This would not require additional experiments but may open other avenues of quantitative and computational comparisons/validations.

• The authors persist in the use of ‘transcriptionally distinct’ as a modifier for their cluster assignments. This should be toned down even more. ‘Distinct implies they are clearly segregated in transcriptional space.’ The silhouette scores provide some additional evidence that the cells are ‘appropriately clustered’ but does not indicated that for example, adjacent cells in the UMAP space that are annotated as separate clusters are truly ‘distinct’ from each other. These (as well as most cellular subtypes) exist on continua. Clustering is fine for analytical purposes, but care should be taken when identifying/calling ‘distinct’ cellular subtypes.

• The comparison of DAprog-1 to RG cells is a rich addition, and their lack of similarity is intriguing. It would be nice if the authors might speculate on why these differences exist (e.g. if DAprog-1 is not a _proliferating_ progenitor does it represent a more immature DA neuron subtype arising from specific culture conditions?). This is a loose request, I understand, but it seems germane to the discussion of ‘pseudotemporal’ progression of DA neurons in the 3D culture system.

© 2022. Published by The Company of Biologists under the terms of the Creative Commons Attribution License (https://creativecommons.org/licenses/by/4.0/).

Second revision

Author response to reviewers' comments

Reviewer 1 Advance Summary and Potential Significance to Field:
This paper provides useful and interesting methods to culture organoids of human fetal dopamine neurons. These methods, will no doubt, be of interest to the field.

Reviewer 1 Comments for the Author:
The authors have taken on board the vast majority of comments from the reviewers and I commend them for their diligence and the production of a more refined manuscript.
I am pleased with the revised version, with one exception.

The title suggests that these are organoids of fetal "ventral midbrain". However, the fact that this cannot be verified by direct comparison to authentic fetal ventral midbrain, and the fact that many differences are likely evident between the organoids and authentic midbrain (ie loss of Sert+ neurons, loss of astrocytes in the organoids), it is not accurate to call these "organoids of ventral midbrain".
They are, instead, something more akin to organoids of human fetal dopamine neurons.

We are happy that the reviewer is please with the revision made.
Based on the remaining comment we have now titled the manuscript: “Single-cell transcriptional and functional analysis of dopaminergic neurons in organoid-like cultures derived from human fetal midbrain”.

Reviewer 2 Advance Summary and Potential Significance to Field:
The study by Birtele et al profiles transcriptional signature of individual cells from the first trimester developing human ventral midbrain, revealing profiles for floor plate progenitors, radial glia, early dopaminergic, serotonergic/glutamatergic neurons, and oculomotor/trochlear nucleus neurons as well as progenitors and non-neural cells. Because these cells are difficult to access in the second trimester, this study establishes 2D and 3D culture conditions to extend their maturation finding that the 3D approach preserves functional DA neurons.

This study is significant for providing a new resource of single cell gene expression in clinically-relevant cell types that are difficult to access, for providing an approach for studying human DA neurons ex vivo as they mature, potentially including primate-specific populations not present in mouse, and for exploring culture methods that support new grafting strategies.

Reviewer 2 Comments for the Author:
The authors have mainly responded to the suggestions raise. I have largely minor comments
We are happy for the overall positive view of the revised version of the manuscript and also thank the reviewer for the remaining comments and suggestions that we have addressed as outlined below.

Major comments:
•Line 165 - “at an accelerated pace as would be expected during normal development in the intact fetal VM.” I am unsure what the authors mean by “accelerated pace” - is there empirical data that the rate of change in maturation properties exceeds that expected from earlier stages (or in vivo)? If not, then the word accelerated is not appropriate. And if the pace would expected from in vivo development, then that would also not be accelerated.

We based that sentence on the fact that DA neurons mature over a long time period in vivo, but we did not make a direct comparison in this study. We understand that the sentence can be confusing and have therefore removed it.

Minor comments:
•Line 24 - change to “This approach revealed”

Now modified in the text.

© 2022. Published by The Company of Biologists under the terms of the Creative Commons Attribution License (https://creativecommons.org/licenses/by/4.0/).

•Line 60 “have” should be “has”

Now modified in the text.

•Lane 80 - explain abbreviation LSM

Now modified in the text.

•Line 101 change to “canonical markers”

Now modified in the text.

•Line 135 – TH-low appears to express canonical intermediate progenitor markers like HES6, DLL1, GADD45G, INSM1, NHLH1, NEUROG2

Now inserted in the text.

•Line 143-149 and figure 1K and 1L - there's some mislabeling and the colors have been swapped. The neuroblast cluster is called Prog and the Serotonergic cluster is labeled Glut/Sert N rather than Sert> Maybe wrong version of figure? Fig 1D-E and Supplementary 1F-G have the same labeling and colors.

Figure 1D-E is now updated. Now all the panels have similar labeling and colors.

•Line 151-153 - I personally still think the TH high and low division might be attributed to maturity but I think we can leave this alone

We briefly mention this as the two groups show difference in channels composition.

•Figure 3D - new image of GIRK2 but not very clear. Maybe show a zoomed in inset without DAPI?

We have now inserted an inset without DAPI for both CALB and GIRK2 images.

•Line 192 and figure 3E - new panel in figure 3 could have it's own letter (3F)

Now modified and inserted the new figure as 3F.

•Line 194 – 198 – thank you for adding these helpful references!
•Line 234 – more clarification of the exact criteria for substetting the 12,600 cultured DA neurons would be important and also a defense of using the word “mature” here as less than half of them express TH. Was this actually subsetting the DA lineage vs mature DA neurons?

We apologize for the lack of clarity. Analyzed cells included both immature and mature dopaminergic neurons. We have now specified this and included it in the text.

•Line 247-251 - this new sentence is a bit unlcear to me and I'm not sure I understand correctly - I don't really see NR4A2 expression in DAProg-1 based on figure 4I-J

DAProg-1 did show a limited level of NR4A2 as seen in the heatmap of fig 4I light green, absence of expression would be indicated by yellow color.

•Line 299 should clarify, as in the rebuttal that CALB1 and GEM do not appear to be localized to a particular subset of DA neurons

We have now clarified this point.

•Line 373 – not sure that Kanton 2019 shows meaningful overlap of maturation signatures with adult brain snRNA-seq vs limited preservation of regional and differentially expressed markers
We have removed the mentioned citation.

© 2022. Published by The Company of Biologists under the terms of the Creative Commons Attribution License (https://creativecommons.org/licenses/by/4.0/).

Reviewer 3 Advance Summary and Potential Significance to Field:
- Paper is improved from previous version in many, but not all places.
- Specific suggestions are made below that might help to resolve authors' stated inability to respond to specific requests from reviewers.

• Response generally _acknowledges_ concerns of the reviewers but in a few cases does not _address_ the concerns directly.
• Additional validation and comparison(s) to human VM DA neurons still not as deep as I would like (suggesting that in lieu of smFISH which is a _very_ difficult ask, that authors compare their data a bit more deeply to Wang et al 2022 preprint data; link below)
• Authors have generally taken recommendations for increased clarity and for toning down conclusions to heart.

We thank the Reviewer for their generally positive comments on the work re-presented and happy that the revisions clarified most issues. We have addressed remaining issues as described below.

Specific Response Points:
• The addition of the WGCNA module analysis, as requested, is commendable, however, little 'biological' interpretation has been made from/with this analysis. Other than identifying GO terms with enrichment in these modules (relatively few of which appear to be relevant or contextually related to DA neurons or their roles/development) the authors provide only a cursory description of how the learned components are 'used' in the in vivo dataset. No quantification is described, and no discussion about the biological interpretations of what individual modules represent about the comparative similarities/differences between DA-subtypes in vitro and in vivo. The result here is that the specific analysis was technically 'performed' but no meaningful value was extracted from the results (which was the original intent from the reviewer's ask).

WGCNA analysis showed that fetal tissue cultured in 3D exhibited a defined and distinct molecular signature compared to non-cultured fetal tissue. Furthermore, GO enrichment in Transmission Chemical Synapse terms shown by the blue module points to the fact that DA neurons emerging from 3D culture showed more mature molecular features. 3D fetal culture allows us to increase our understanding of the later stages of DA neurogenesis, which are otherwise difficult to investigate due to severely limited access to fetal tissue after the second trimester. We have now more clearly insreted these points in the new Results section on page 9.

•The specific request for a differential comparison to the in vivo DA neurons (Argwal 2020) is a solid addition, demonstrating primarily that the largest differences in gene expression might simply be attributed to differences in maturation rate.

We are grateful to the Reviewer for their positive feedback.

• The authors indicate that a direct comparison of DA-1/2 subtypes with A9-A10 subtypes is not possible due to low data quality/availability. Specifically, the authors reply that "[a] pre-press paper by Wang et al., recently published in biorXiv which reports a scRNAseq dataset from human DA neurons in the substantia nigra.
Unfortunately, the authors did not release the raw data needed to perform a direct comparative analysis between our fetuses and their adult DA neuron subclusters." While not directly mentioned in the specified preprint, a quick online search indicated that these data are in fact available on the NIH SRA (see link and project accession below). Given the quality, breadth, and depth of these comparable in vivo data, this should make response to the specific request (better mapping of DA-1 and Da-2 subtypes to in vivo subtypes) possible. I leave the requirement for this comparison at the discretion of the authors and editors.

NOTE: We have removed unpublished data that had been provided for the referees in confidence.

© 2022. Published by The Company of Biologists under the terms of the Creative Commons Attribution License (https://creativecommons.org/licenses/by/4.0/).

• Given the authors' (completely understandable) response that availability of human fetal tissue for smFISH or other validation is severely limited, and the authors' assertions that their focus " in this manuscript specifically aims to generate a transcriptional profile of the developing human fetal VM and functionally mature human DA neurons, proposing 3D fetal culture as a model to authentically recapitulate the later stages of DA differentiation…", I would suggest that a further comparison to the high-depth/quality Wang et all 2022 preprint data would be sufficient additional (and external) validation. Especially if performed at the DA-neuron subtype level. This would not require additional experiments but may open other avenues of quantitative and computational comparisons/validations.

Please, see the comment above

• The authors persist in the use of 'transcriptionally distinct' as a modifier for their cluster assignments. This should be toned down even more. 'Distinct implies they are clearly segregated in transcriptional space.' The silhouette scores provide some additional evidence that the cells are 'appropriately clustered' but does not indicated that for example, adjacent cells in the UMAP space that are annotated as separate clusters are truly 'distinct' from each other. These (as well as most cellular subtypes) exist on continua. Clustering is fine for analytical purposes, but care should be taken when identifying/calling 'distinct' cellular subtypes.

We have now rephrased the sentence according to the Reviewer´s suggestion (page 7, line 234) and modified accordingly throughout the text.

• The comparison of DAprog-1 to RG cells is a rich addition, and their lack of similarity is intriguing. It would be nice if the authors might speculate on why these differences exist (e.g. if DAprog-1 is not a _proliferating_ progenitor, does it represent a more immature DA neuron subtype arising from specific culture conditions?). This is a loose request, I understand, but it seems germane to the discussion of 'pseudotemporal' progression of DA neurons in the 3D culture system.

This is a really important and interesting point, and we thank the Reviewer for raising it. Unfortunately, due to the limited number of fetal samples, we could not provide a reliable pseudotime analysis to reconstruct the developmental trajectories of the identified DA subclusters. However, the data obtained so far does allow us, at this point, to make some hypotheses. It is possible to speculate that the DA prog-1 cluster may be an intermediate immature cell type from which both or only one of the detected mature DA neuron clusters are specified. We plan to address this issue in the future by collecting and analyzing other fetal samples. Our lab is, in fact, currently running a sister project, where we transplanted fetal cells dissected from fetal VM tissues into a PD rat model in order to analyze the specification and diversification of DA neurons at single-cell resolution also *in vivo*.
We believe that integrating both *in vivo* and *in vitro* datasets in the future will help us to i) better define the signature of the DA clusters detected, ii) reconstruct their developmental trajectories, and iii) get some novel insights into one of the central questions in the field: whether DA neuron subtypes are specified during development or emerge only after the post- natal stage.

© 2022. Published by The Company of Biologists under the terms of the Creative Commons Attribution License (https://creativecommons.org/licenses/by/4.0/).

Third decision letter

MS ID#: DEVELOP/2022/200504

MS TITLE: Single-cell transcriptional and functional analysis of dopaminergic neurons in organoid-like cultures derived from human fetal midbrain

AUTHORS: Marcella Birtele, Petter Storm, Yogita Sharma, Janko Kajtez, Jenny Nelander Wahlestedt, Edoardo Sozzi, Fredrik Nilsson, Simon Stott, Xiaoling L He, Bengt Mattsson, Daniella Rylander Ottosson, Roger A. Barker, Alessandro Fiorenzano, and Malin Parmar
ARTICLE TYPE: Research Article

I am happy to tell you that your manuscript has been accepted for publication in Development, pending our standard ethics checks.

© 2022. Published by The Company of Biologists under the terms of the Creative Commons Attribution License (https://creativecommons.org/licenses/by/4.0/).
